# From the Sunlit to the Aphotic Zone: Assembly Mechanisms and Co-Occurrence Patterns of Protistan-Bacterial Microbiotas in the Western Pacific Ocean

**DOI:** 10.1128/msystems.00013-23

**Published:** 2023-02-27

**Authors:** Ping Sun, Ying Wang, Yifan Zhang, Ramiro Logares, Peng Cheng, Dapeng Xu, Bangqin Huang

**Affiliations:** a State Key Laboratory of Marine Environmental Science, Xiamen University, Xiamen, China; b Key Laboratory of the Ministry of Education for Coastal and Wetland Ecosystem, Xiamen University, Xiamen, China; c Fujian Province Key Laboratory for Coastal Ecology and Environmental Studies, College of the Environment and Ecology, Xiamen University, Xiamen, China; d Institute of Marine Sciences, CSIC, Barcelona, Catalonia, Spain; e Institute of Marine Microbes and Ecospheres, College of Ocean and Earth Sciences, Xiamen University, Xiamen, China; Max Planck Institute for Marine Microbiology

**Keywords:** aphotic community, protists, bacteria, deterministic and stochastic processes, microbial associations

## Abstract

We know little about the assembly processes and association patterns of microbial communities below the photic zone. In marine pelagic systems, there are insufficient observational data regarding why and how the microbial assemblies and associations vary from photic to aphotic zones. In this study, we investigated size-fractionated oceanic microbiotas, specifically free-living (FL; 0.22 to 3 μm) and particle-associated (PA; >3 μm) bacteria and protists (0.22 to 200 μm) collected from the surface to 2,000 m in the western Pacific Ocean, to see how assembly mechanisms and association patterns changed from photic to aphotic zones. Taxonomic analysis revealed a distinct community composition between photic and aphotic zones that was largely driven by biotic associations rather than abiotic factors. Aphotic community co-occurrence was less widespread and robust than its photic counterparts, and biotic associations were crucial in microbial co-occurrence, having a higher influence on photic than aphotic co-occurrences. The decrease in biotic associations and the increase in dispersal limitation from the photic to the aphotic zone affect the deterministic-stochastic balance, leading to a more stochastic-process-driven community assembly for all three microbial groups in the aphotic zone. Our findings significantly contribute to our understanding of how and why microbial assembly and co-occurrence vary from photic to aphotic zones, offering insight into the dynamics of the protistan-bacterial microbiota in the western Pacific’s photic and aphotic zones.

**IMPORTANCE** We know little about the assembly processes and association patterns of microbial communities below the photic zone in marine pelagic systems. We discovered that community assembly processes differed between photic and aphotic zones, with all three microbial groups studied (protists and FL and PA bacteria) being more influenced by stochastic processes than in the photic zone. The decrease in organismic associations and the increase in dispersal limitation from the photic to the aphotic zone both have an impact on the deterministic-stochastic balance, resulting in a more stochastic process-driven community assembly for all three microbial groups in the aphotic zone. Our findings significantly contribute to the understanding of how and why microbial assembly and co-occurrence change between photic and aphotic zones, offering insight into the dynamics of the protist-bacteria microbiota in the western Pacific oceans.

## INTRODUCTION

Microbial organisms are critical components of marine ecosystems, and their activity influences biogeochemical cycles in a range of marine habitats, either directly or indirectly (1, 2). Bacteria are the most abundant microbial organisms in the oceans. In the upper ocean, they can reach densities of approximately 10^6^ mL^−1^, where they consume roughly half of the daily primary production (3). Bacterial communities are typically divided into free-living (FL) and particle-associated (PA) bacteria by size filtration, despite dynamic exchanges between the two communities (4). Due to differences in chemistry and physics between particles and surrounding marine waters (5), particles host microbial communities distinct from those in surrounding waters (6). Protists are a large complex grouping of primarily unicellular eukaryotic organisms that are negatively defined as not belonging to the animal, fungal, or plant kingdom (7). They are a crucial component of the microbial food web and function in a variety of ecological niches as primary producers, predators, symbionts or parasites (1, 2). For instance, photosynthetic protists are the foundation of the marine food web and serve as a source of nutrition for other organisms (8). Heterotrophic protists are the major predators of bacteria and small eukaryotes, and their predation or grazing is essential for carbon and energy transfer to higher trophic levels (9) and for the release of dissolved nutrients to the base of microbial food webs (1). Moreover, protists and prokaryotes form symbiotic relationships across the functional spectrum from facultative to obligate and from mutualistic to parasitic (10, 11). However, it is uncertain to what extent these diverse groups of species interact with one another and are affected by environmental variation.

A central question in ecology is how microbial communities are assembled (12). Uncovering this information would facilitate the understanding and prediction of how the microbial community varies with space and time (13). A unified conceptual framework for the assembly of microbial communities was established (14), which is determined by a combination of deterministic and stochastic ecological processes. Deterministic processes are primarily characterized by selection exerted by biotic and abiotic factors. Biotic variables, such as predation, competition, mutualism, and tradeoffs, can impact community assembly, whereas abiotic factors, such as temperature, salinity, and pH, can exert selection on microbial communities through environmental filtering (15). Stochastic processes primarily reflect random variations in species’ relative abundance, and dispersion events, birth, death, and drift all have the potential to affect community assembly (16). Historically, the majority of research on microbial community assemblies has focused on environmental selection, that is, on the impact of abiotic factors, while the roles of biotic factors have been considered less frequently (10). Protist-bacterium associations are widespread in the marine environment (17), encompassing a diverse variety of interdependencies that either are stable or change over time (18). Heterotrophic protists, such as ciliates and heterotrophic flagellates, are known to graze on bacteria in the oceans, dramatically altering the community structure, activity, and physiological state of their prey (19). Additionally, research has revealed symbiotic relationships between bacteria and protists (20). For example, in the tropical oligotrophic North Atlantic Ocean, the loricated ciliate *Codonella* has ectobiotic cyanobacterial symbionts (21). Furthermore, it has been observed that protists and bacteria are involved in the interaction between vital trace elements, micronutrients, and macronutrients (22). Phytoplankton exudates, for instance, have been proven to be a crucial carbon source for bacterial growth (23) and to play a substantial role in nitrogen and phosphate mineralization (24). Bacteria provide phytoplankton with growth promoters (25), vitamins (or vitamin precursors) (26), and other nutrients, including vibrioferrin (27) and methylamine (28). The community composition of predator, host, and nonpredatory microbial alliances (as determined by the exchange of important elements, such as vitamins) is apparently a significant underlying factor influencing the assembly of bacterial communities. However, protists and bacteria have rarely been investigated simultaneously as an organismal factor to reveal the role of biotic interactions in community assembly studies (29, 30), especially in the marine aphotic zones (31, 32).

The underlying mechanisms governing microbial community variation have been demonstrated in oceanic waters. The analysis of community turnover on a large spatial scale indicates that selection and dispersal limitation appear to be the primary ecological processes that shape communities (29, 33). For example, protist co-occurrence in the global oceans and neotropical rainforest soils is largely determined by selection and dispersal limitation (33). Picoeukaryotic communities are primarily governed by dispersal limitation, whereas bacterial communities are shaped by dispersal limitation, selection, and ecological drift in the surface layer of the tropical and subtropical global ocean (29). However, few studies have examined the mechanisms by which the microbial community in the aphotic zone of the oceans, and even fewer have examined both protistan and bacterial communities (34, 35). A survey of size-fractionated prokaryotic communities from the surface down to 4,000 m in the Atlantic, Pacific, and Indian Oceans revealed that community variation is primarily determined by selection and dispersal events (35). A time series observation of bacterial communities at the Bermuda Atlantic Time-series Study site (BATS) revealed that selection was the most critical ecological process governing communities in both the surface and the 200-m layer (34). To date, the mechanisms underlying the assembly of aphotic microbial communities remain poorly understood, owing to the lack of research on more assemblages and the low-resolution depth sampling of microbial data. To address the processes governing the distribution of microbial communities and to further elucidate the generality of protistan and bacterial community assembly mechanisms from photic to aphotic zones, studies involving both domains of microbial life (protists and bacteria) and dense depth layer sampling conducted at the same spatial scales are required.

With these considerations in mind, we investigated simultaneously the assembly and association of protistan and FL and PA bacterial communities collected from 6 to 11 depth layers from the surface down to 2,000 m in the western Pacific Ocean (see Fig. S1A in the supplemental material), using high-throughput sequencing of the V3 and V4 regions of the 16S and 18S rRNA genes, respectively. This sample design enabled us to gain a better understanding of how and why protistan-bacterial assembly and association vary from photic to aphotic zones in marine pelagic systems, shedding light on the general assembly mechanisms between assemblages from photic to aphotic zones. The ocean is vertically structured, with differentiation occurring due to differences in temperature and salinity between water layers. Additionally, the sampling area is influenced by complex water masses (36), which are more weakly mixed in deep waters than in shallow waters (Fig. S1B to H). As a result, we hypothesize that changes in water density differences and the movement of water masses form barriers preventing water mixing, resulting in increased dispersal limitation and, consequently, greater stochastic impacts on aphotic microbial communities. In addition, given the widespread interaction of protists and bacteria in the oceans and the importance of predator and host community composition for bacterial community structure, we expect that protistan-bacterial interactions through the multiple, complex relationships would result in similar complex co-occurrence networks across different water depth layers.

10.1128/msystems.00013-23.1 FIG S1 (A) Map of the Western Pacific Ocean with field sampling stations highlighted. (B) Potential temperature (θ, in degrees Celsius) versus salinity for the sampling stations. The colored dots indicate the different sampling stations. (C to H) Temperature, salinity, and DO profiles versus depth (C to E, 0 to 500 m; F to H, 0 to 2,000 m) for the sampling stations. The colored lines represent the sampling stations, while the colored circles represent the sampling depths. Download FIG S1, TIF file, 5.4 MB. Copyright © 2023 Sun et al. 2023 Sun et al. https://creativecommons.org/licenses/by/4.0/ This content is distributed under the terms of the Creative Commons Attribution 4.0 International license.

## RESULTS

### Differences in community composition between photic and aphotic zones.

**FIG 1:**
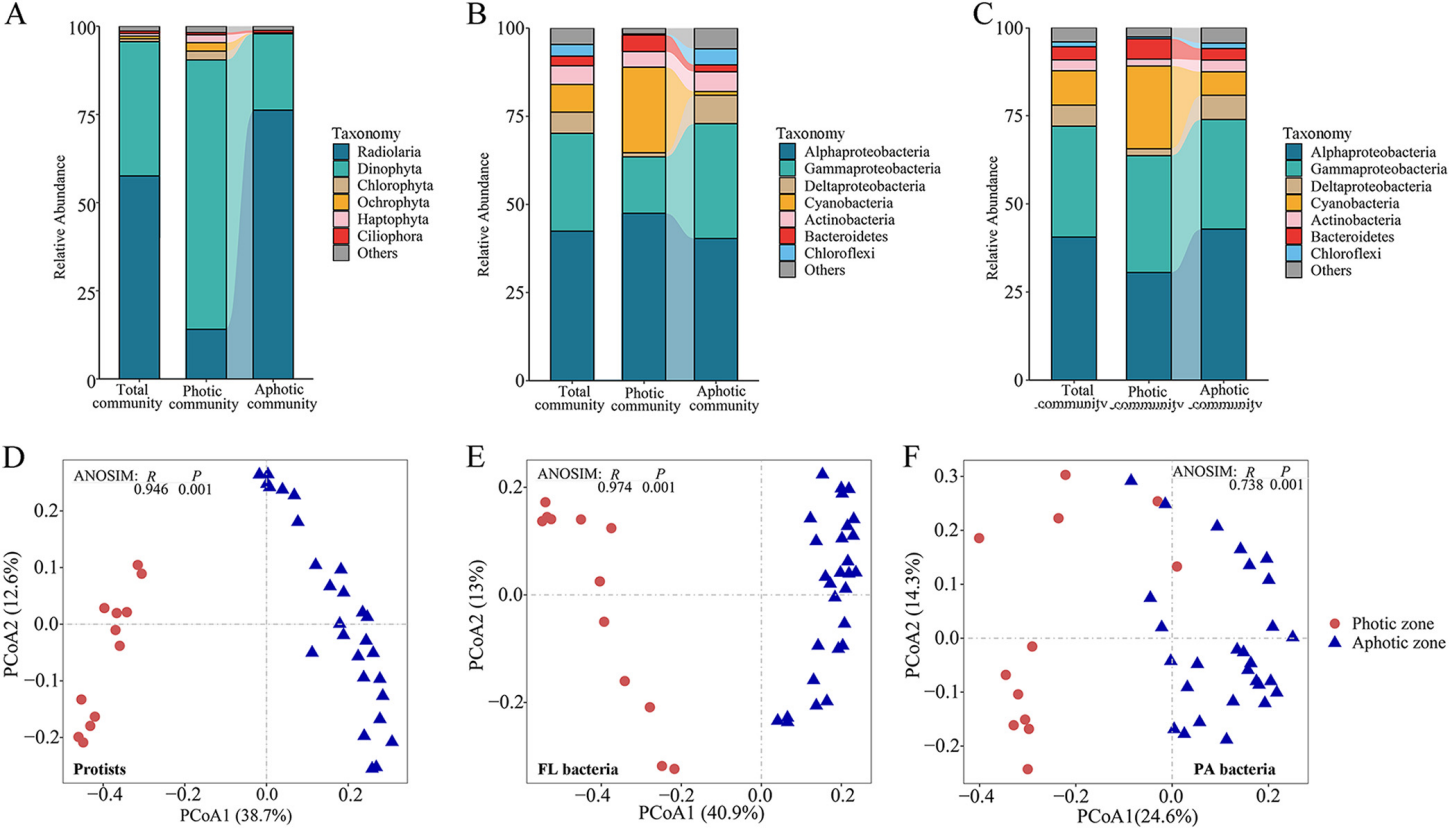
(A to C) Bar plots illustrating the protistan (A), FL bacterial (B), and PA bacterial (C) composition for the total, photic, and aphotic communities. (D to F) PCoA illustrating community dissimilarities of protists (D), FL bacteria (E), and PA bacteria (F). All three microbial communities were separated into photic and aphotic clusters (ANOSIM: *P < *0.001).

We investigated the protistan, FL bacterial, and PA bacterial communities separately in 40 samples collected from the surface to 2,000 m to investigate the distribution, assembly, and association of protistan-bacterial microbiotas in the photic and aphotic zones of the western Pacific (Fig. S1; Table S1). Overall, we observed significant spatial differences in microbial community compositions in all three microbial groups from the photic to the aphotic zones, as determined by analysis of similarities (ANOSIM) (Fig. 1). About 95% of the protistan sequences were assigned to Radiolaria (57%) and Dinophyta (38%), highlighting the primary role of the two protistan assemblages in the water columns of the western Pacific (Fig. 1A). Dinophyta were significantly enriched in the photic zone (76% in the photic zone) and significantly reduced in relative abundance in the aphotic zone (21% in the aphotic zone). The Radiolaria exhibited the opposite pattern (14% in the photic zone versus 76% in the aphotic zone). Other assemblages of protists contributed less than 1%. *Alphaproteobacteria* and *Gammaproteobacteria* made up 70% to 72% of the total bacterial communities (Fig. 1B). *Alphaproteobacteria* were considerably more abundant in photic FL bacterial communities than in aphotic FL bacterial communities, but *Gammaproteobacteria* exhibited the opposite trend. Nonetheless, *Alphaproteobacteria* and *Gammaproteobacteria* exhibited an inverse distribution trend from photic to aphotic zones in PA bacterial communities (Fig. 1C). *Cyanobacteria* constituted 7.8% to 9.7% of overall bacterial communities, being more prevalent in photic (23.4% to 24.2%) than aphotic (1.0% to 6.6%) zones. Other assemblages, such as Deltaproteobacteria, *Actinobacteria*, *Bacteroidetes*, and *Chloroflexi*, are minor in the total bacterial communities.

10.1128/msystems.00013-23.6 TABLE S1 Sampling information for waters collected from the western Pacific Ocean. Download Table S1, DOCX file, 0.02 MB. Copyright © 2023 Sun et al. 2023 Sun et al. https://creativecommons.org/licenses/by/4.0/ This content is distributed under the terms of the Creative Commons Attribution 4.0 International license.

### Depth decay of community similarity.

We employed principal-coordinate analysis (PCoA) to visualize the clustering of microbial communities and discovered that protistan, FL bacterial, and PA bacterial communities were divided into two distinct depth clusters: photic and aphotic communities (Fig. 1D to F). ANOSIM results confirmed that photic and aphotic communities were significantly distinct (*P < *0.001), which was consistent across all three microbial communities, with global *R* values ranging from 0.738 to 0.974 (Fig. 1D to F). Then, we examined the variations in the protistan, FL bacterial, and PA bacterial communities along a depth gradient (from surface to 2,000 m). We used a simple linear regression to plot the Bray-Curtis dissimilarity indices (i.e., relative abundance based and presence-absence based) versus the depth distance between the water samples (Fig. S2). The pattern of depth decay was significant (*P* < 0.001), and goodness-of-fit statistics were high (*R*^2^ = 0.70 to 0.73 for protists; *R*^2^ = 0.65 to 0.66 for FL bacteria), indicating that community similarity varied significantly with water depth in protists and FL bacteria (Fig. S2A and D). The slope of the FL bacterial community was steeper than that of the protistan community, indicating that FL bacterial community turnover was more rapid than that of protists along a depth gradient (Fig. S2A and D). PA bacteria also exhibited the depth decay pattern, but with a much lower goodness-of-fit statistic (*R*^2^ = 0.21 to 0.22) than the other two microbial groups (Fig. S2A and D). Analyses of photic and aphotic microbial communities revealed that the depth decay pattern was stronger in bacteria than in protists in the photic zone but reversed in the aphotic zone and that the depth decay pattern was generally stronger in the aphotic zone than the photic zone (Fig. S2B, C, E, and F).

10.1128/msystems.00013-23.2 FIG S2 Relationship between microbial community similarity and depth distance in total (A and D), photic (B and E), and aphotic (C and F) communities as determined by linear regression analysis. The relative abundance-based (A to C) and presence-absence-based (D to F) Bray-Curtis dissimilarity indices were employed to determine community similarity, and Euclidean distance was used to generate similarity matrices for depth distance. Download FIG S2, TIF file, 3.1 MB. Copyright © 2023 Sun et al. 2023 Sun et al. https://creativecommons.org/licenses/by/4.0/ This content is distributed under the terms of the Creative Commons Attribution 4.0 International license.

### Drivers and assemblies of protistan-bacterial microbiotas.

**FIG 2:**
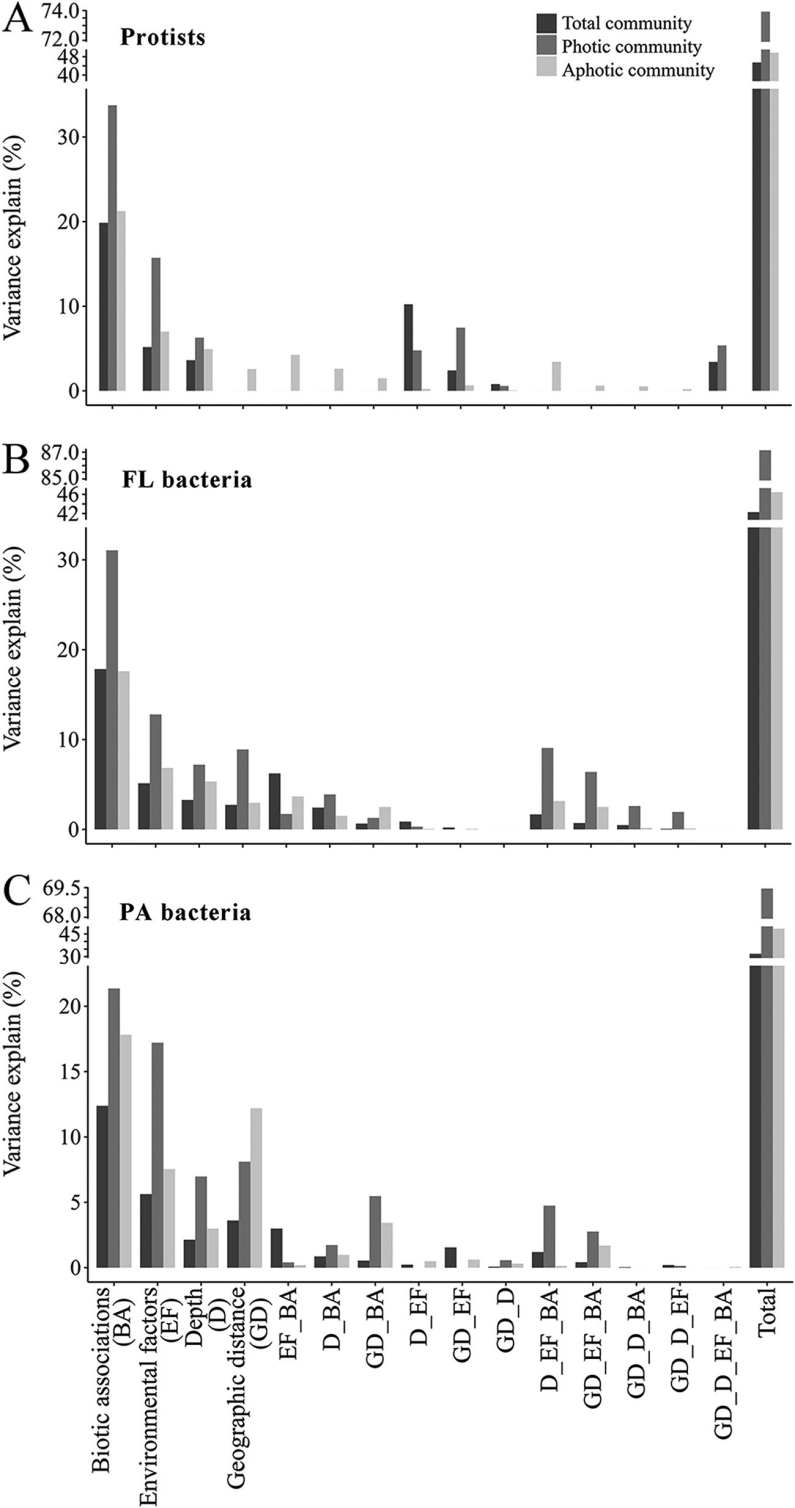
(A to C) Contributions of spatial variables, biotic associations and environmental factors to the dissimilarities of protistan (A), FL bacterial (B), and PA bacterial (C) communities, as determined by variation partitioning analysis. The bar plots depict the amount of variation in the protistan (A), FL bacterial (B), and PA bacterial (C) communities that can be explained by a given single matrix (D, depth; GD, geographic distance; BA, biotic associations; EF, environmental factors) or a given combination of matrices, as indicated on the *x* axis.

We employed variation partitioning analysis (VPA) to explore the factors driving the community vertical distribution, since a distinct community composition was observed between the photic and aphotic zones. All variables were classified into four categories (depth, geographic distance, biotic association, and environmental variables), and VPA was performed for each microbial data set (Fig. 2). In all cases, biotic association was the strongest predictor and explained about 12% to 20% of the overall variance. When the effects of each variable were separated, biotic association remained the strongest predictor for all microbial communities (Fig. 2). Additionally, other variables, such as environmental variables, depth, and geographic distance, were significant predictors of total community variation. When the drivers of photic and aphotic communities were examined, it was found that biotic association had a significant effect on microbial communities. However, the influence of biotic association decreased when the zone transitioned from photic (21.4% to 33.7%) to aphotic (17.6% to 21.2%) (Fig. 2). The pure effect of environmental variables revealed a similar trend, with the variance explained by environmental variables ranging from 12.8% to 15.7% of photic communities to 6.8% to 7.5% of aphotic communities. In comparison to biotic associations and environmental variables, the relative contribution of spatial variables was generally greater in aphotic than photic zones, with variance explained by spatial variables ranging from 6.3% to 16.1% in photic communities to 7.5% to 15.2% in aphotic communities (Fig. 2). The fraction of unexplained variance in all three microbial groups tends to increase when the zone transitions from photic to aphotic, owing primarily to the lower explained fractions by biotic associations in aphotic zones (Fig. 2).

**FIG 3:**
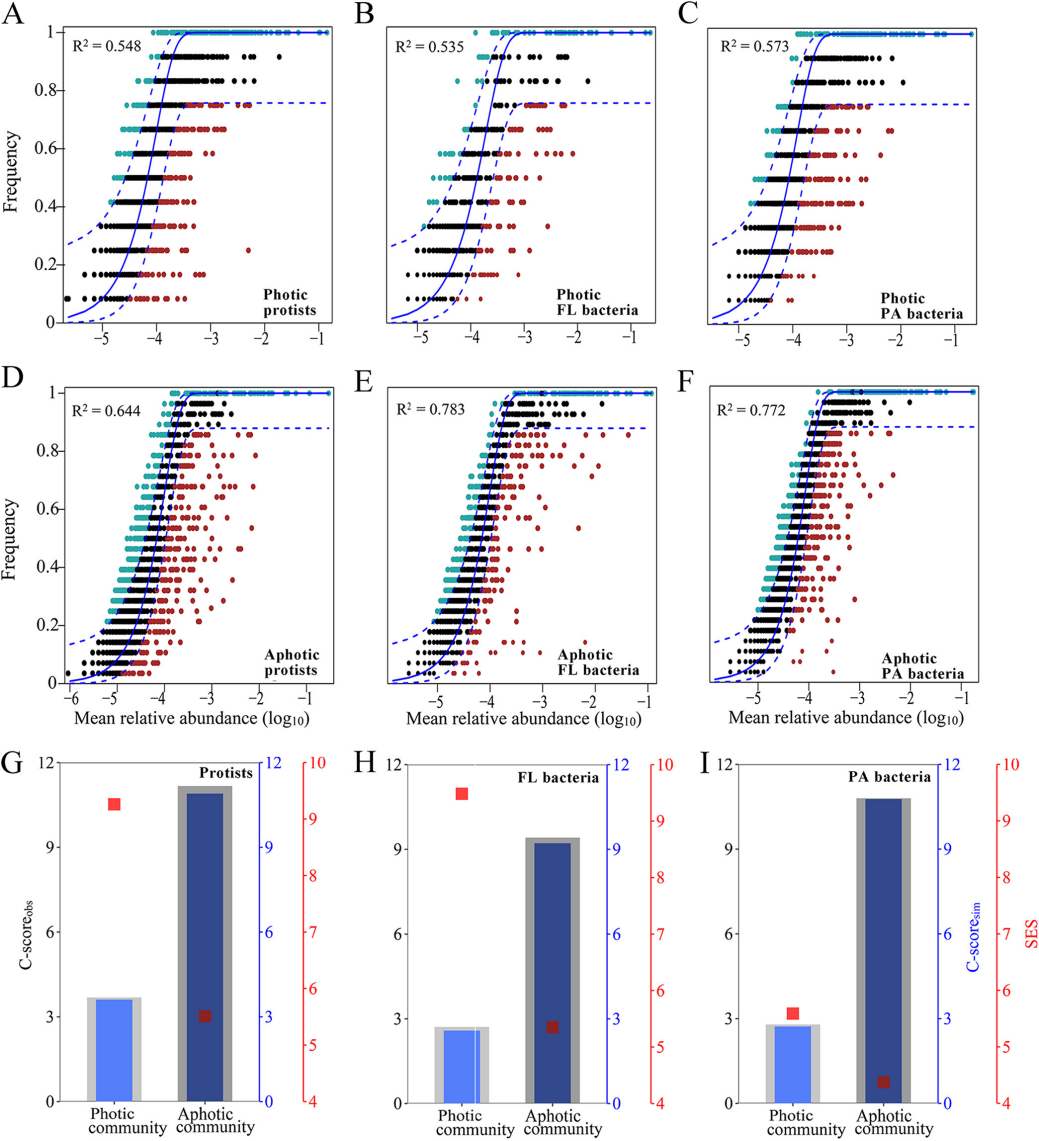
(A to I) Fitting Sloan’s neutral model from photic to aphotic zones for protists (A and D), FL bacteria (B and E), and PA bacteria (C and F). (G to I) The C-score metric is calculated using null models for photic and aphotic protistan (G), FL bacterial (H), and PA bacterial (I) communities. The observed C score (C-score_obs_) is greater than the simulated C score (C-score_sim_), indicating the presence of nonrandom patterns of co-occurrence. Standardized effect sizes less than and greater than −2 and −2 denote aggregation and segregation, respectively.

We employed Sloan’s neutral model to determine how the protistan, FL bacterial, and PA bacterial communities are assembled. Overall, the frequency with which microbial taxa occurred in individual communities was relatively well described by the neutral model (Fig. 3A to F). However, the fit of the model varied with the zone transition from photic to aphotic, with the goodness-of-fit (*R*^2^) values increasing from 0.535 to 0.573 in photic communities to 0.644 to 0.783 in aphotic communities (Fig. 3A to F). The habitat niche breadth, as determined by Levin’s niche breadth index, and the dispersal ability of microbial communities, as determined by the average of pairwise shared sequence numbers of each operational taxonomic unit (OTU), were further estimated to help explain the effects of environmental filtering (deterministic processes) and dispersal limitation (stochastic processes) on microbial community assembly. The average niche breadth was significantly lower for photic than for aphotic communities, which was consistent across all three microbial groups, implying that aphotic microbial communities are less influenced by environmental filtering (Fig. S3A to F). Except for PA bacteria, the average dispersal ability of photic communities was generally greater than that of aphotic communities, indicating that aphotic communities were more dispersal limited (Fig. S3G to L). Furthermore, the checkerboard score (C score), which was used to determine the relative importance of deterministic processes for community assembly, showed that the standardized effect size (SES) decreased from photic to aphotic zones for all three microbial groups (Fig. 3G to I), indicating that deterministic processes were less important in aphotic microbial communities. The results of phylogenetic null model analysis, which distinguishes selection from dispersal events, validated these findings, demonstrating that the importance of dispersal events relative to selection increased in all three groups, as the zones transitioned from photic to aphotic (Table S2). Taken together, these observations indicate that stochastic processes had a greater influence on aphotic communities than on photic ones.

10.1128/msystems.00013-23.3 FIG S3 (A to C and G to I) Violin plots illustrating the habitat niche breadth (*B*_com_) and the average of pairwise shared proportion of sequence numbers of each OTU (a proxy for dispersal ability) in photic and aphotic zones for protistan (A and G), FL bacterial (B and H), and PA bacterial (C and I) communities. The black dots indicate the mean value of *B*_com_ and dispersal ability, and the error bars denote the standard deviation. (D to F and J to L) Density graphs illustrating the distribution of the mean value of *B*_com_ and dispersal ability of protistan (D and J), FL bacterial (E and K), and PA bacterial (F and L) communities after aphotic samples were subsampled 1,000 times to the same number of photic samples. The dotted lines denote the mean value of *B*_com_ and the dispersal ability of photic microbial communities. The Wilcoxon rank-sum test was used to measure the significance of the difference between photic and aphotic communities. ***, *P* < 0.001. Download FIG S3, TIF file, 5.4 MB. Copyright © 2023 Sun et al. 2023 Sun et al. https://creativecommons.org/licenses/by/4.0/ This content is distributed under the terms of the Creative Commons Attribution 4.0 International license.

10.1128/msystems.00013-23.7 TABLE S2 Phylogenetic null model-based mapping of ecological processes in protistan, FL bacterial, and PA bacterial communities in photic and aphotic zones. Download Table S2, DOCX file, 0.01 MB. Copyright © 2023 Sun et al. 2023 Sun et al. https://creativecommons.org/licenses/by/4.0/ This content is distributed under the terms of the Creative Commons Attribution 4.0 International license.

### Distinct co-occurrence networks in photic and aphotic zones.

**FIG 4:**
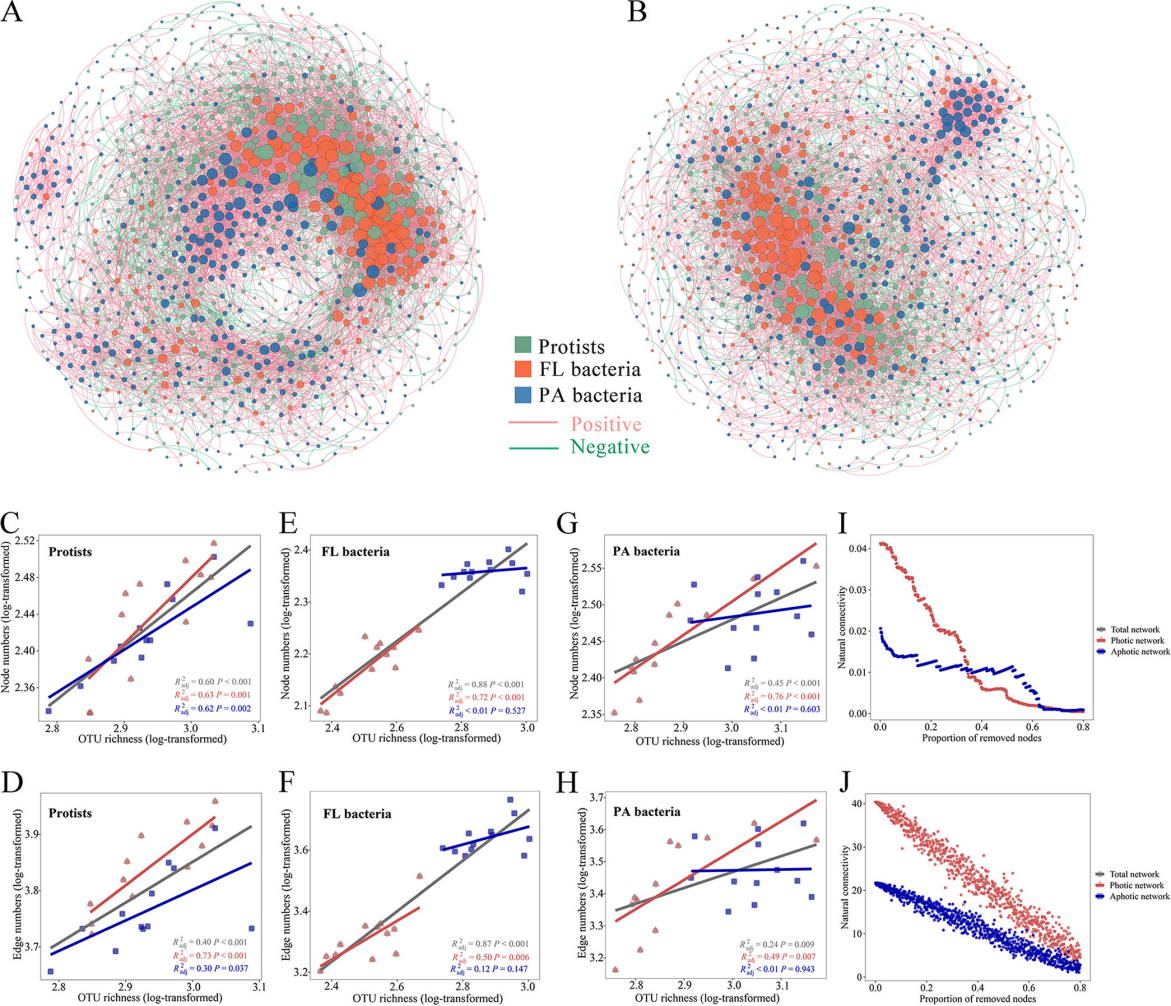
(A and B) Co-occurrence patterns of protist-bacterium microbiota in photic (A) and aphotic (B) zones. A connection indicates a strong and significant correlation (|*r*| ≥ 0.6; FDR-corrected *P* values < 0.01). Positive connections are in red, and negative connections are in cyan. The size of each node is proportional to the degree of the OTUs. Nodes are colored for each of the three assemblages, i.e., protists, FL bacteria, and PA bacteria. (C to H) OTU richness is positively connected with increasing network complexity in terms of network size (nodes) and network connectivity (edges) in protists (C and D), FL bacteria (E and F), and PA bacteria (G and H) across depth layers. (I and J) Network stability was measured for photic and aphotic communities using a network attacking scenario in which nodes were gradually removed in a predetermined order (I) or randomly (J).

Given the importance of biotic associations in determining community variation, we constructed co-occurrence networks (photic and aphotic networks) of protistan and bacterial communities to further elucidate the potential role of biotic associations (Fig. 4A and B). All networks exhibited scale-free properties, as all network degrees followed a power law distribution, indicating that the network structures were not random (Table S3). We combined a linear regression analysis with a method for deciphering taxon-taxon-environment covariates one by one from the network to elucidate the effect of biotic interaction and niche sharing on microbial co-occurrence. Microbial diversity has been used to examine the effects of biotic interactions on the patterns of microbial co-occurrence. As a result, we correlated alpha diversity (a proxy for cross-group biotic interaction) and environmental variables with network size and network connectivity, i.e., node and edge numbers, in order to determine the relative importance of potential interactions and niche sharing in microbial co-occurrence (Fig. 4C to H; Fig. S4; Table S4). Increased network size (number of nodes) and connectivity (number of edges) were found to be significantly correlated with increased alpha diversity, with stronger correlations occurring more frequently in photic than aphotic networks (Fig. 4C to H). There was also a significant correlation between network complexity and environmental variables such as temperature and salinity, but the correlations were generally weaker than those for alpha diversity (Fig. 4C to H; Fig. S4; Table S4). Additionally, by individually testing each network edge for environmental factors, we observed 1,596 and 1,110 taxon-taxon-environment linkages for photic and aphotic networks, respectively, accounting for approximately 19.5% to 21.3% of the networks. Taken together, the data show that potential biotic interactions were more important than environmental factors measured in determining microbial co-occurrence, and their influence was greater on photic than aphotic microbial communities.

10.1128/msystems.00013-23.4 FIG S4 Phylogenetic diversity is positively connected with increasing network complexity in terms of network size (nodes) and network connectivity (edges) in protists (A and B), FL bacteria (C and D), and PA bacteria (E and F) across depth layers. Correlations with extremely low *R*^2^ values (<0.01) were omitted. Download FIG S4, TIF file, 5.7 MB. Copyright © 2023 Sun et al. 2023 Sun et al. https://creativecommons.org/licenses/by/4.0/ This content is distributed under the terms of the Creative Commons Attribution 4.0 International license.

10.1128/msystems.00013-23.8 TABLE S3 Topological properties of the empirical co-occurrence networks and associated random networks in photic and aphotic zones. Download Table S3, DOCX file, 0.01 MB. Copyright © 2023 Sun et al. 2023 Sun et al. https://creativecommons.org/licenses/by/4.0/ This content is distributed under the terms of the Creative Commons Attribution 4.0 International license.

10.1128/msystems.00013-23.9 TABLE S4 Regression coefficient between environmental variables and network size (nodes) and network connectivity (edges). Download Table S4, DOCX file, 0.02 MB. Copyright © 2023 Sun et al. 2023 Sun et al. https://creativecommons.org/licenses/by/4.0/ This content is distributed under the terms of the Creative Commons Attribution 4.0 International license.

The aphotic network was markedly different from the photic network (Fig. 4A and B; Table S3). The average degree, network density, and average clustering coefficient were all lower in aphotic communities, implying that they were less connected (Table S3). The average path length was longer in the aphotic network than in the photic network, implying less tight links between aphotic communities (Table S3). Additionally, we examined the stability of photic and aphotic networks by simulating a network attack scenario (see Materials and Methods for details). With the removal of critical nodes with a high betweenness, photic networks lose connectedness more slowly than aphotic networks (Fig. 4I and J). The random attacking setting demonstrated a similar trend: the natural connectivity of the photic network was consistently higher than that of the aphotic network during the increasing, random removal of nodes, demonstrating the higher robustness of the photic network (Fig. 4I, J). To summarize, photic communities formed a more connected and robust network than aphotic ones.

**FIG 5:**
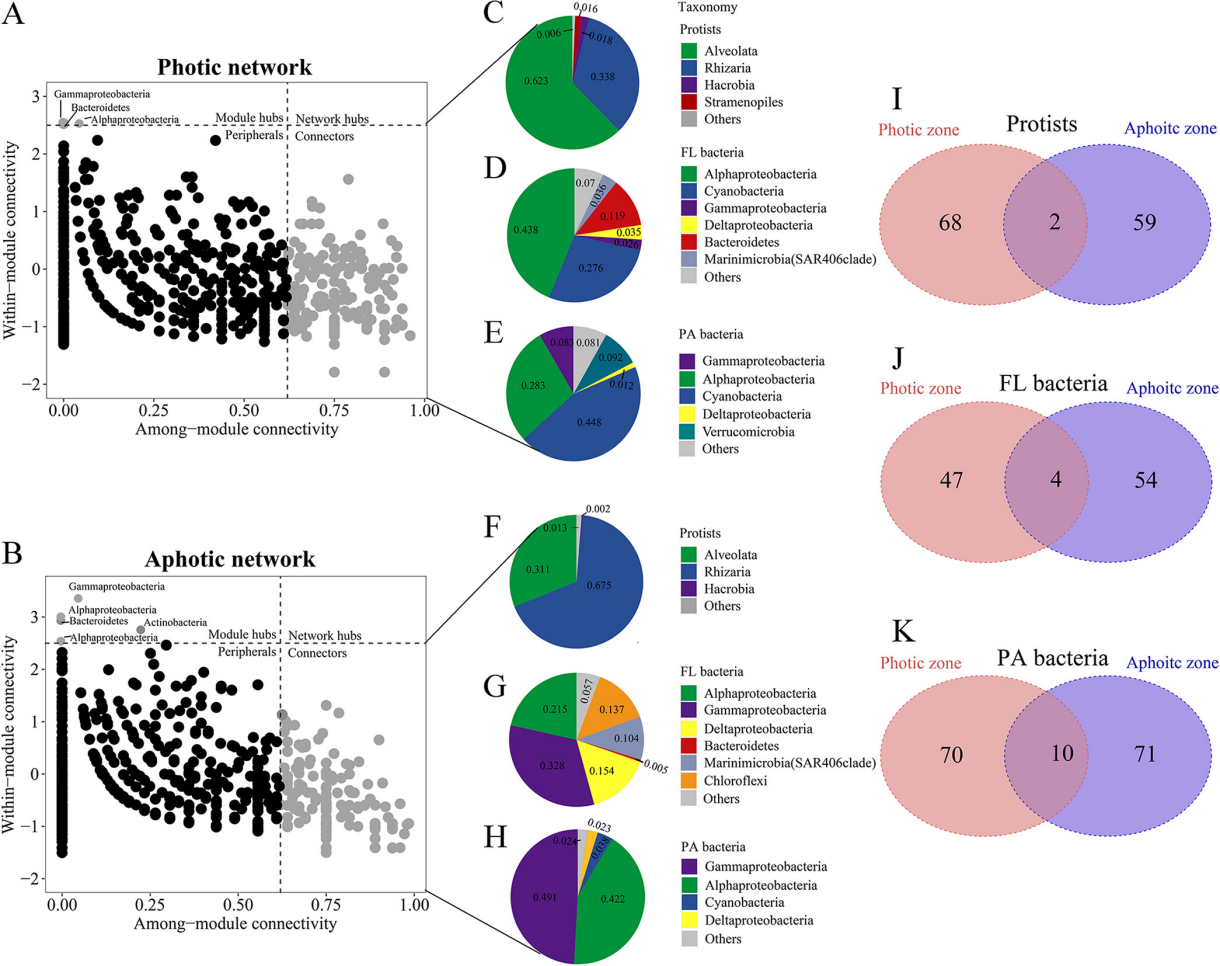
(A and B) Node classification in photic (A) and aphotic (B) networks to identify putative keystone taxa. (C to H) Connector composition in photic and aphotic networks for protists (C and F), FL bacteria (D and G), and PA bacteria (E and H). (I to K) Venn diagrams depicted the number of unique and shared keystone nodes between photic and aphotic networks for protists (I), FL bacteria (J), and PA bacteria (K).

Additionally, we identified possible keystone taxa in photic and aphotic networks by categorizing nodes into four groups (network hubs, module hubs, connectors, and peripherals; see Materials and Methods for details) based on their within-module (*Z_i_*) and among-module (*P_i_*) values (Fig. 5A and B). Due to their critical significance in network topology, module hubs and connectors have been identified as keystone taxa. According to these criteria, members of Alveolata (protists), *Alphaproteobacteria* (FL bacteria), and *Cyanobacteria* (FL and PA bacteria) would be the most prominent keystone taxa in the photic networks (Fig. 5C to E). Rhizaria (protists), *Gammaproteobacteria* (FL and PA bacteria), and *Alphaproteobacteria* (FL and PA bacteria) would be the most prominent keystone assemblages in aphotic networks, as they accounted for more than half of the relative abundance of connectors in the aphotic network (Fig. 5F to H). Notably, only 4.15% of all keystone nodes were shared throughout photic and aphotic networks, implying that keystone taxa were not conserved at the node level during the transition from photic to aphotic zones (Fig. 5I to K; Table S5). When photic and aphotic networks are combined, they support a comparable number of keystone species, yet only a few taxa at the node level serve as keystones for both photic and aphotic networks; however, taxa from higher taxonomic levels do recur between photic and aphotic networks.

10.1128/msystems.00013-23.10 TABLE S5 Keystone OTUs identified in both photic and aphotic networks. Download Table S5, DOCX file, 0.01 MB. Copyright © 2023 Sun et al. 2023 Sun et al. https://creativecommons.org/licenses/by/4.0/ This content is distributed under the terms of the Creative Commons Attribution 4.0 International license.

## DISCUSSION

### Microbial structure and co-occurrence in photic and aphotic zones.

Clone libraries, fingerprints, high-throughput sequencing, and metagenomics have revealed differential patterns of bacterial communities that occur in photic versus aphotic zones in a vast array of marine environments (35, 37–45). Observational data on the vertical distribution of FL and PA bacteria are abundant, whereas data on protists, particularly below the photic zone, are lacking (32, 45, 46). Similar to the bacterial community, the protistan community in the present study was clearly stratified down the water column, demonstrating a large vertical difference between photic and aphotic protistan-bacterial microbiomes in the western Pacific. Recent studies, including those on protistan communities at station ALOHA in the North Pacific Subtropical Gyre (45), in the subtropical and subantarctic waters of the southwest Pacific (46), and in the tropical and subtropical global oceans (32), support this conclusion. The dichotomy in distribution of protists and bacteria along the water column observed in the present study and at both local and global scales likely underlies the heterogeneity of biotic, abiotic, and dispersal conditions encountered in photic and aphotic zones, and this variation in biotic, abiotic, and dispersal conditions may be a crucial factor that can directly influence community composition.

Simultaneous observations for protistan, FL bacterial, and PA bacterial communities allow us to determine the relative importance of organismic associations as a biotic factor in the assembly of microbial communities and how it varies between photic and aphotic zones. The present study discovered that biotic associations were the strong predictors of community variations and co-occurrence networks in the photic and aphotic zones (Fig. 2 and 4), demonstrating the importance of biotic associations in shaping protistan-bacterial communities in the water column of the western Pacific. Historically, it was believed that abiotic variables had a stronger impact on the organization of microbial communities; however, there has recently been a greater awareness of biotic influences (47). Biotic associations are ubiquitous in oceanic waters; competition, predation, and mutualism can modify community structure and co-occurrence pattern by maintaining distinct taxa (17). Similar to our findings, cross-domain biotic factors (protists and bacteria) were found to be crucial in shaping protistan and bacterial communities in the European freshwater environment (48) and in the river-influenced coastal upwelling system in the South China Sea (30). A recent study discovered that microbial associations were not distributed randomly on the network and were driven by both local and global patterns, indicating that biotic interaction is essential in shaping microbial co-occurrence in the global oceans (10).

Our findings revealed not only the significance of biotic association in microbial communities but also a reduced effect on aphotic communities compared to photic communities. Moving from the photic to the aphotic zones, the gradual decrease in light inhibits photosynthesis, resulting in significantly lower cell densities than those observed in the photic zone (49, 50). Both protistan and bacterial cell abundances decrease from photic to aphotic zones (49–52), which could result in a reduced influence of biotic associations on microbial communities due to fewer possibilities for interaction. Compared to protists and FL bacteria, the influence of biotic associations on community variation in PA bacteria was relatively small (Fig. 2). One probable explanation is that PA bacteria thrive in particle environments that are abundant in nutrients, particularly carbon (53). Thus, PA bacteria were copiotrophic rather than oligotrophic (10). The increased nutritional opportunities in the particle environment may help to alleviate species competition. This scenario would loosen the relationship between community composition and biotic variables, allowing stochastic processes to exert a greater influence.

The network topological parameter, the average clustering coefficient, was much higher in the photic network than in the aphotic network (Table S3), implying that the photic network is more complicated. Due to network buffering, high-complexity networks typically have a higher degree of stability (54). As a result, it is likely that the photic network was more stable than the aphotic network. This is validated by the network attacking method, which demonstrated that the photic network was more robust than the aphotic network as key/random nodes were removed. The photic zone is subjected to fluctuations caused by sunlight, tides, wind, and current movement. The more dynamic and heterogeneous environment in the photic zone (environmental homogeneity test: *F* = 36.466, *P* < 0.001) may result in a greater number of environmental variables to which photic microbes can respond. It is expected that stronger environmental filtering and the greater microbial interactions in the photic zone would favor fast-responding microbes over slow-responding ones, resulting in a more connected and stable photic network than the aphotic network. Due to the unmeasured environmental variables, such as nutrient concentration, which could also influence the co-occurrence of microbial communities (55), and the fact that correlation in correlation-based networks does not always indicate causation/interaction, these co-occurrence links in the network should be regarded as putative biotic interactions.

### Assembly of microbial communities from photic to aphotic zones.

The present study revealed a more stochastic process-driven community assembly for all three microbial groups in the aphotic zone, which was supported by the three approaches (i.e., the neutral model, the null model, and the phylogenetic null model) used to infer the assembly mechanisms of microbial communities. There are several possible explanations. First, the decreased biotic associations and a less dynamic environment in the aphotic zone may reduce the importance of deterministic processes in the aphotic community assembly. Second, aphotic communities exhibited greater niche breadths than photic communities for all three microbial groups (Fig. S3A to F). Given that microorganisms with broader niches may be less influenced by the environment, aphotic communities were probably less influenced by environmental filtering. Third, the weaker mixing of deep waters relative to shallow waters in our sampling area may result in a greater dispersal limitation and, as a result, greater stochastic impacts on aphotic microbial communities. Based on plots of temperature, salinity, and dissolved oxygen against depth, the waters of all sampling sites mixed to some extent above 110 m, while the deep waters were clearly separated into roughly two groups in the deep zones (Fig. S1B to H). Additionally, the dispersal ability of photic microbial communities was considerably greater than that of aphotic microbial communities when all three microbial groups were examined independently (Fig. S3). Regression analyses revealed a slight negative association of dispersal ability with depth and geographic distance, but a significantly stronger negative correlation with water masses (Fig. S5), demonstrating that spatial factors are less important in dispersal limitation than the movement of water masses. In addition, we observed that the dispersal of PA bacteria was greater in the aphotic zone than in the photic zone. The majority of our aphotic samples were collected from the mesopelagic zone, where substantial remineralization of organic matter occurred (56). A recent study on mesopelagic bacteria revealed a strong negative correlation between prokaryotic carbon losses and species richness, because fragmentation of fast-sinking into nonsinking particles and active cell detachment may enrich the surrounding nonsinking prokaryotic fractions, thereby increasing their overall species richness (57). Therefore, the consumption of particulate matter by bacteria in the mesopelagic zone would result in the transformation of large particulate matter into numerous small particles, facilitating the dispersal of PA bacteria.

10.1128/msystems.00013-23.5 FIG S5 Relationship between the average of pairwise shared proportion of sequence numbers of each OTU (a proxy for dispersal ability) and spatial factors/water mass (Euclidean distance of temperature, salinity, and DO was used to generate dissimilarity matrices for water mass distance) for protistan (A to C), FL bacterial (D to F), and PA bacterial (H to J) communities, as determined by linear regression analysis. Download FIG S5, TIF file, 3.9 MB. Copyright © 2023 Sun et al. 2023 Sun et al. https://creativecommons.org/licenses/by/4.0/ This content is distributed under the terms of the Creative Commons Attribution 4.0 International license.

### Conclusions.

**FIG 6:**
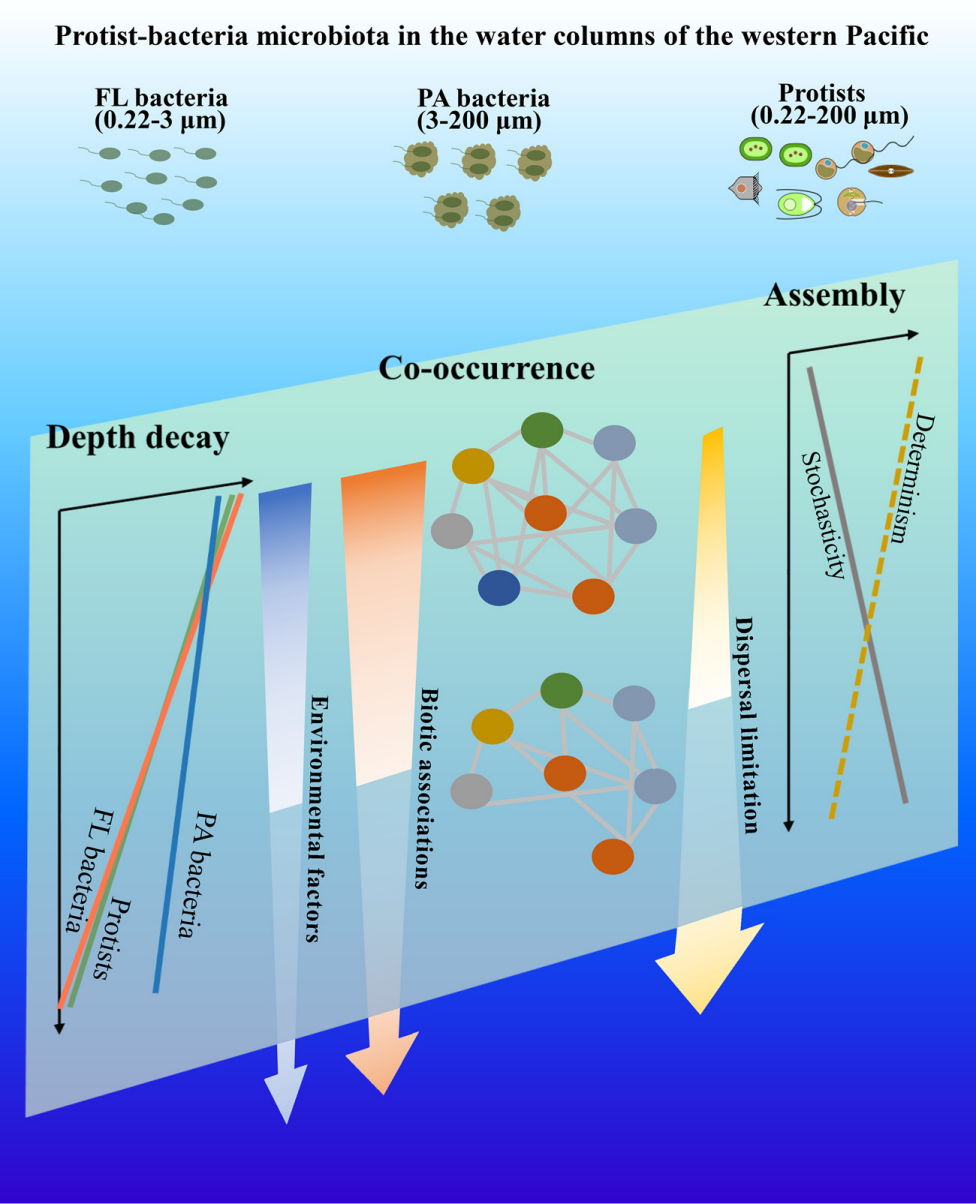
A conceptual framework for community variation, assembly mechanisms, and co-occurrence patterns in the protist-bacteria microbiota, which are essentially determined by biotic associations and dispersal limitation.

We present a conceptual framework to characterize the variation, assembly, and co-occurrence of protistan and bacterial communities from the surface to a depth of 2,000 m in marine pelagic systems (Fig. 6). Protists, FL bacteria, and PA bacteria exhibited distinct compositions between the photic and aphotic zones, with biotic associations rather than abiotic factors being determinative of the differences. Between the photic and aphotic zones, microbial co-occurrence exhibited distinct network structures that became less widespread and less robust, and biotic associations were a critical variable with a greater impact on photic co-occurrence than on aphotic co-occurrence. Decreased biotic associations and the increased dispersal limitation from the photic to aphotic zones affect the deterministic-stochastic balance, resulting in a more stochastic process-driven community assembly for all three microbial groups in the aphotic zone. Additionally, the difference in network complexity between photic and aphotic zones results in a shift in the distribution of the potential keystone species, with only <5% of them present in both photic and aphotic networks. Future research on the assembly and co-occurrence of microbial communities must therefore integrate both biotic and abiotic factors.

## MATERIALS AND METHODS

### Sample collection and environmental factor analyses.

Our field sampling area is located in the western North Pacific Ocean (130°E, 2.25°N to 18°N) (Fig. S1A; Table S1). A total of 120 samples (40 samples each for protists, FL bacteria, and PA bacteria) were collected aboard the R/V Kexue at discrete water depths (i.e., 5 m, deep chlorophyll maximum [DCM], 200 m, 500 m, 1,000 m, and 2,000 m for three sampling sites (E130-7, E130-13, and E130-28), as well as more intense vertical sampling efforts for two sites (ME-3 and ME-14), i.e., 5 m, 75 m, DCM, 150 m, 200 m, 300 m, 500 m, 600 m, 800 m, 1,000 m, and 2,000 m) from 6 October to 7 November 2018 (Fig. S1A; Table S1). Fifteen liters of seawater was prefiltered using 200-μm nylon mesh (Sefar Nitex) to eliminate mesozooplankton and then filtered sequentially through polycarbonate filters with pore sizes of 3 μm and 0.22 μm (Millipore, USA). The FL and PA bacterial communities were collected using water samples with filters with pore sizes of 0.22 μm and 3 μm, respectively. The protistan community was collected using a combination of 0.22-μm- and 3-μm-pore-size filters. The filters were immediately flash-frozen in liquid nitrogen and stored at −80°C until further processing in the laboratory.

Depth, temperature, salinity, and dissolved oxygen (DO) concentrations were measured *in situ* with a Sea-Bird conductivity-temperature-depth (CTD) profiler (SBE 917; Sea-Bird Electronics, USA). Bacterial and viral abundance analysis followed (58). Seawater (1.8 mL) was prefiltered with a 20-μm mesh, mixed with ice-cold glutaraldehyde (1% final concentration) for 15 min in the dark, and then flash-frozen in liquid nitrogen and stored at −80°C. Bacterial and viral abundances were analyzed using a flow cytometer (Beckman Coulter; Epics Altra II) with a 306C-5 argon laser (Coherent, Santa Clara, CA, USA). For bacterial abundance analysis, samples were thawed at 37°C in the laboratory and then stained with SYBR green I (1/10,000 final concentration) in the dark for 15 min at room temperature. Then, 10 μL fluorescent microspheres (diameter of 1 μm; 10^5^/mL; Molecular Probes, Eugene, OR, USA) was added to the 1 mL dyed samples as an internal standard. The samples were run at a flow rate of 0.1 to 1 mL/h. The enumeration of bacterial abundance followed (59). Heterotrophic bacteria were identified in the plots of red fluorescence versus green fluorescence. For viral abundance analysis, samples were thawed at 37°C and diluted 5 to 50 times with Tris-EDTA buffer (pH 8; Sigma-Aldrich). Then, the samples were stained with SYBR green I (1/20,000 final concentration) and heated at 80°C for 10 min in the dark, and cooled for 5 min before analysis. The samples were run at a flow rate of 0.1 to 1 mL/h. The enumeration of viral abundance followed (60). The viruses were identified based on the green fluorescence and side scatter signal. Abundance analysis of nanoflagellates, including heterotrophic nanoflagellates (HNFs) and pigmented nanoflagellates (PNFs), followed (61). A 50-mL seawater sample was prefiltered with 20-μm nylon mesh to remove large plankton, fixed with ice-cold glutaraldehyde at a final concentration of 1%, and stained with DAPI (4′,6-diamidino-2-phenylindole; Sigma-Aldrich, St. Louis, MO, USA) for 10 min. The abundance of HNFs and PNFs was determined using epifluorescence microscopy (Olympus, Japan).

### DNA extraction, PCR amplification, and sequencing.

DNA was extracted according to the manufacturer’s instructions using the AllPrep DNA/RNA minikit (Qiagen, United States). The hypervariable region V3-V4 of the protistan 18S rRNA and the bacterial 16S rRNA genes were amplified using eukaryote-specific primers (F1, 5′-CCA GCA SCY GCG GTA ATT CC-3′; R3, 5′-ACT TTC GTT CTT GAT YRA-3′) (62) and bacterium-specific primers (341F, 5′-CCT AYG GGR BGC; 806R, 5′-GGA CTA CNN GGG TAT CTA AT-3′) (63). PCR conditions followed those described in references 62 and 63. It should be noted that haptophytes, a significant assemblage of protists, may be underestimated when these universal primer sets are used, due to the fact that a single base mismatch on the 3′ end of the reverse primers and their GC-rich genomes may impede amplification (64). Each sample was amplified in triplicate, pooled, and purified using the Wizard SV gel and PCR clean-up system kit (Promega, USA). All purified fragments were sequenced on the Illumina MiSeq PE300 sequencing platform by MajorBio Bioinformatics Technology Co., Ltd. (Shanghai, China).

### Sequence data processing and statistical analyses.

Cleaning of raw sequence data was carried out with Trimmomatic v.0.38 (65) and Flash v.1.2.11 (66), following the criteria in reference 67. The following criteria had to be met. (i) Low-quality reads with an average quality score below 20 and a read length below 50 bp were eliminated. (ii) Reads containing ambiguous characters and mismatches in the barcode or primer were eliminated. (iii) Reads with an overlapped region of less than 10 bp or a mismatch ratio greater than 0.2 were deleted. Chimera and singleton removal and OTU clustering were carried out with USEARCH v10 (68). Representative sequences were clustered with a 97% sequence similarity following (29). The protistan reads were classified against the Protist Ribosomal Reference database v4.11.11 (PR2) (69) using the command “sintax” with default settings in USEARCH v10. For bacterial reads, taxonomy was assigned against the SILVA v132 database (70) using the command “assign_taxonomy.py” in QIIME (BLAST method with default setting). All reads classified as nonprotists for the protistan data set and nonbacteria for the bacterial data set were removed. Before further analysis, OTU tables of protistan and FL and PA bacterial communities were normalized by randomly resampling to 35,480, 25,124, and 25,124 reads per sample, corresponding to the lowest sequence count in protistan and bacterial data sets, respectively. The profiling of the protistan community yielded 3,099,476 high-quality sequences (range, 35,515 to 136,573; median, 78,079), which corresponds to 3,281 protistan OTUs. The profiling of the FL bacterial community resulted in the identification of 2,201,904 high-quality sequences (range, 33,428 to 94,273; median, 49,263), which corresponds to 3,795 FL bacterial OTUs. The profiling of the PA bacterial community yielded 2,249,543 high-quality sequences (range, 25,124 to 93,605; median, 52,091), which corresponds to 5,097 PA bacterial OTUs.

### Microbial distribution pattern.

The microbial community distribution was displayed using principal-coordinate analysis (PCoA) based on Bray-Curtis dissimilarities, which converts data on distances between samples into map-based visualization of those samples, facilitating exploring dissimilarities of samples. Analysis of similarity (ANOSIM) which is a robust nonparametric test for differences in resemblances among groups of samples was employed to examine differences in microbial communities between photic and aphotic zones. Depth decay analysis was performed with a linear least-squares regression on the relationship between depth distance versus microbial community similarity (i.e., relative abundance-based and presence-absence-based Bray-Curtis dissimilarity indices). We used variation partitioning analysis (VPA), which divides the variation of a response variable (here, community variation) among different explanatory data sets (here, spatial, biotic and abiotic variables) to partition the community variation (pairwise Bray-Curtis dissimilarity) into spatial, biotic, and abiotic effects. Community variation was partitioned into effects of four categories: depth, geographic distance (spatial variables derived from geographic distances using Moran’s eigenvector maps), biotic association (the first two axes of microbial PCoA ordination and principal-component analysis [PCA] ordination of biotic factors, i.e., abundances of bacteria, virus, HNF, and PNF according to the Kaiser-Guttman rule), and environmental effects (the first two axes of physiochemical PCA ordination according to the Kaiser-Guttman rule) (71). The permutation test was used to assess the significance of each component by partitioning. The *P* values for ANOSIM and VPA were corrected for multiple comparisons using the Bonferroni method. The statistical analyses were carried out using the R packages “stats” (72) and “vegan” (73).

### Community assembly mechanism.

We used the neutral community model (NCM) to evaluate the possible contribution of neutral processes to microbial community assembly (74). The NCM predicts the relationship between the frequency of taxa in a collection of local communities and their abundance within the wider metacommunity (74). Under neutral community assembly, highly abundant taxa should be ubiquitous, since they are more likely to disperse by chance throughout multiple sampling sites, whereas rare taxa should be more likely to be lost in different sites due to ecological drift. For model fitting, we used the approach proposed in reference 16. *R^2^* represents the goodness of fit for the NCM. Calculation of 95% confidence intervals around all fitting statistics was done by bootstrapping with 1,000 bootstrap replicates.

Besides the NCM, we also use the null model (checkerboard score [C score]) to infer the relative importance of deterministic processes on community assembly (75). The C score is calculated as (*R_i_* − *S*) (*R_j_* − *S*) for each pair of species, where *R_i_* and *R_j_* are the total occurrences of species *i* and *j*, respectively, and *S* is the number of core samples containing both species *i* and *j*; this score is then averaged over all possible species pairs. We then calculated the standardized effect size for the C score (SES_c_) by examining the deviation of each observed matrix and simulated null matrix. The SES for the C score was calculated as (*I*_obs_ − *I*_sim_)/SD_sim_, where *I*_obs_ is the observed matrix, *I*_sim_ is the simulated matrices, and SD_sim_ is the standard deviation of the simulated matrices. An SES value that is greater or less than the null values is interpreted as indicating overdispersion or underdispersion, respectively, and the magnitude of the SES is interpreted as the strength of the community’s impact of deterministic processes (76). The C score was calculated using 30,000 simulations with the R package “EcoSimR” (77).

To further differentiate selection from dispersal events, we performed a phylogenetic null model analysis, which quantifies the relative importance of selection and dispersal events on microbial communities (78). The fundamental driving processes, including heterogeneous selection, homogeneous selection, dispersal limitation, homogenizing dispersal, and undominated, were determined by integrating weighted β-nearest-taxon index (βNTI) and RC_bray_ (78). As suggested in reference 78, null models were generated using 1,000 randomizations.

### Community niche breadth and dispersal ability.

To help reveal the influence of deterministic and stochastic processes on protistan and bacterial communities, we estimated niche breadth and dispersal ability at community level in photic and aphotic zones. Levins’ niche breadth (*B*) index was calculated using the “niche.width” function in the package “spaa” (79). The formula is as follows:

$$B_{j}=\frac{1}{\sum_{i=1}^{N}P_{\mathrm{ij}}^{2}}$$

*B_j_* represents the habitat niche breadth of OTU *j* in a metacommunity; *N* is the total number of communities in each metacommunity; and *P_ij_* is the proportion of OTU *j* in community *i*. The average *B* values of all taxa in a single community (*B*_com_) were calculated to indicate habitat niche breadth at the community level. To compare the *B*_com_ of photic and aphotic communities, we randomly subsampled the aphotic samples 1,000 times to the same number of photic samples and recalculated the *B*_com_ of the 1,000 resampled communities.

To estimate the dispersal ability of each taxon that is passively driven by water masses, we followed the method described in reference 71, which calculated the pairwise shared proportion of sequence numbers and used the average shared proportion as a proxy for dispersal. A greater fraction of sequences that are shared indicates that passive movement by water masses was more successful (80). The method we used to estimate microbial dispersal ability may have been influenced by the sample size used. To determine whether the dispersal ability results were biased by the unequal sampling depth, we subsampled the aphotic samples 1,000 times to the same number of photic samples and recalculated the dispersal ability of the 1,000 resampled communities.

### Microbial co-occurrence pattern.

To identify associations among and between protists, FL bacteria, and PA bacteria, co-occurrence networks in photic and aphotic zones were constructed. We constructed microbial networks using the SparCC algorithm, which is well known for its robustness to compositional effects (81). Prior to network construction, the aphotic samples were normalized to the same number as the photic communities using the R function “sample.” To reduce the complexity of the network analysis, OTUs found in less than 30% of samples and with a relative abundance of less than 0.01% were excluded from the analysis. The robust correlations were exported as a GML (Graph Modeling Language) format network file with correlation coefficient |*r*| values of ≥0.6 and false discovery rate-corrected *P* values of <0.01 (82). The interactive programs Gephi v0.9.2 and Cytoscape v3.7.2 were used to visualize networks, perform modular analysis, and determine node-level topological properties (83, 84).

To identify the potential keystone taxa in the networks, each node’s connectivity was determined using its within-module connectivity (*Z_i_*) and among-module connectivity (*P_i_*) (85). Nodes with *Z_i_* values of >2.50 and *P_i_* values of >0.62 were defined as network hubs with a high degree of connectivity both within and between modules. Those with *Z_i_* values of >2.50 and *P_i_* values of <0.62 were defined as module hubs with a high degree of connectivity within a module. Nodes with *Z_i_* values of <2.50 and *P_i_* values of >0.62 serve as connectors among modules. Nodes with *Z_i_* values of <2.50 and *P_i_* values of <0.62 were peripherals with few connections to other nodes. All hubs and connectors could be considered potential keystone taxa due to their critical roles in network structure formation (86). *Z_i_* and *P_i_* were calculated using the Cytoscape plugin GIANT, following reference 85. The formulas are as follows:

$$Z_{i}=\frac{k_{\mathrm{ib}}-k_{b}}{k_{b}}$$

and

$$P_{i}=1-\overset{N_{M}}{\underset{c=1}{\sum }}{\left(\frac{k_{\mathrm{ic}}}{k_{i}}\right)}^{2}$$

where *k_ib_* denotes the number of links of node *i* with other nodes in its module, *k_b_* denotes the average value of within-module connectivity over all the nodes in module *b*, *k_i_* denotes the number of links of node *i* in the entire network, *k_ic_* denotes the number of links from node *i* to nodes in module *c*, and *N_M_* denotes the number of modules in the network.

The robustness of the networks was evaluated by simulating a network attack scenario (87). The approach predetermined the order of nodes based on hub characteristics such as betweenness centrality and degree (87). When nodes in the static network were removed, the loss of natural connectivity was measured and how quickly network robustness degraded was estimated. Continuous loss of natural connectivity will eventually result in the disintegration of a network, but the rate of disintegration will vary depending on the network structure. If the removed node’s associations can be maintained via other nodes, the network is robust to its removal (88).

By examining the relationship between alpha diversity and network topology, we were able to determine the effect of biotic associations on microbial co-occurrence patterns (89). To infer the effect of biotic associations and abiotic factors on microbial co-occurrence, we used linear regression analysis to examine the relationship between biotic associations/environmental factors and network size (node)/connectivity (edge) using the lm function in the “stats” R package (72). Additionally, we used a method described in reference 10 to determine if each network edge was related to environmental factors (i.e., environmental filtering). If an edge between two taxa exists as a result of their covariation with environmental conditions, then there should be strong correlations between each taxon and representative environmental factors. To find taxon-taxon-environment edges, we tested all of the networks’ edges against the environmental variables as described in reference 90.

### Data availability.

All the sequencing sequences for 16S and 18S rRNA genes from this study have been deposited in the public NCBI Sequence Read Archive (SRA) database under BioProject accession number PRJNA663234. The R script used was deposited in GitHub (https://github.com/ying-wang09/mSystems20230106.git).
