# Supplementary figures and images for: From the Sunlit to the Aphotic Zone: Assembly Mechanisms and Co-Occurrence Patterns of Protistan-Bacterial Microbiotas in the Western Pacific Ocean
Source: mSystems. 2023 Feb 27;8(2):e00013-23. doi: 10.1128/msystems.00013-23 (PMC10134807; doi:10.1128/msystems.00013-23)

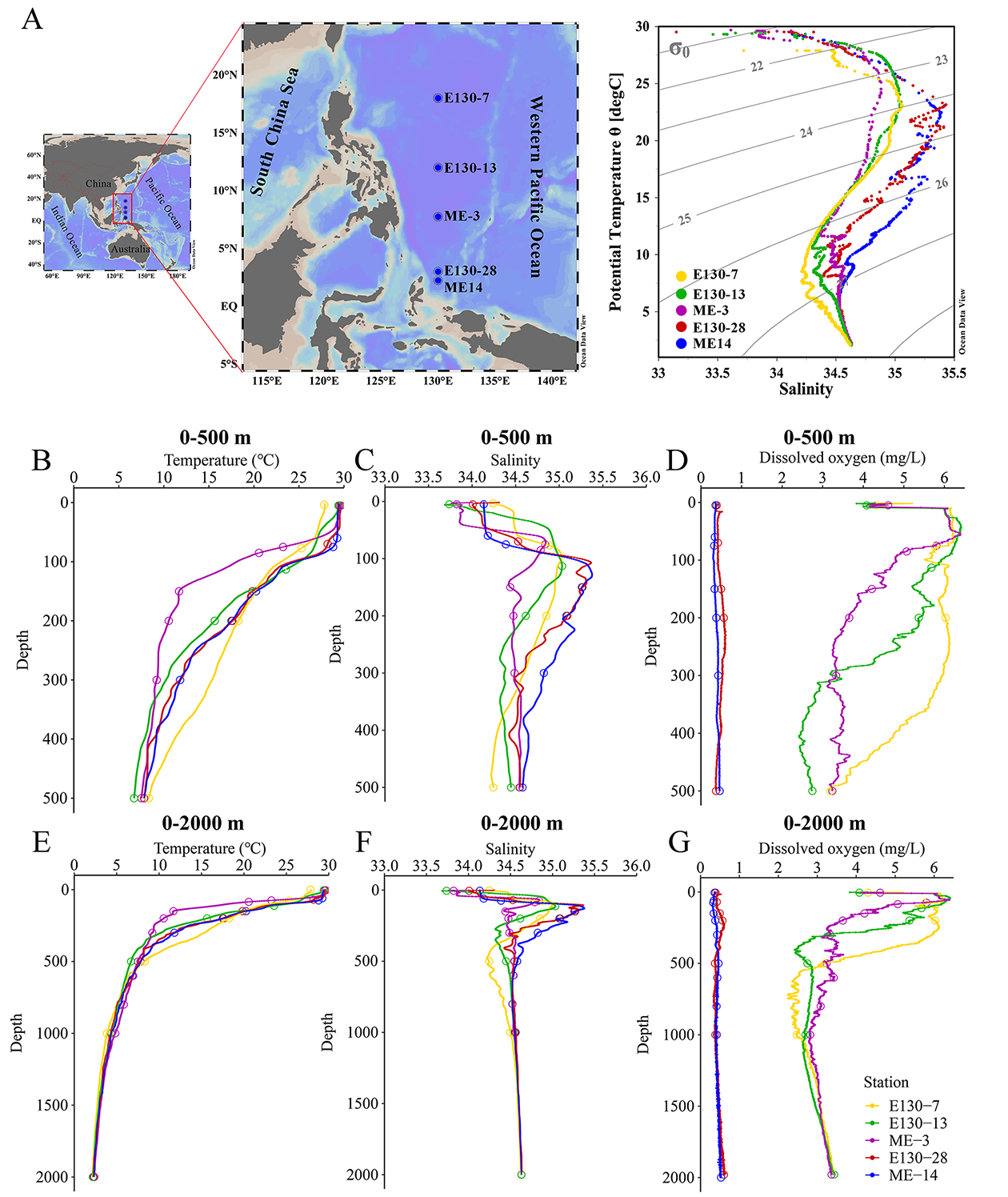

Supplement: FIG S1 [file msystems.00013-23-s0001.tif]

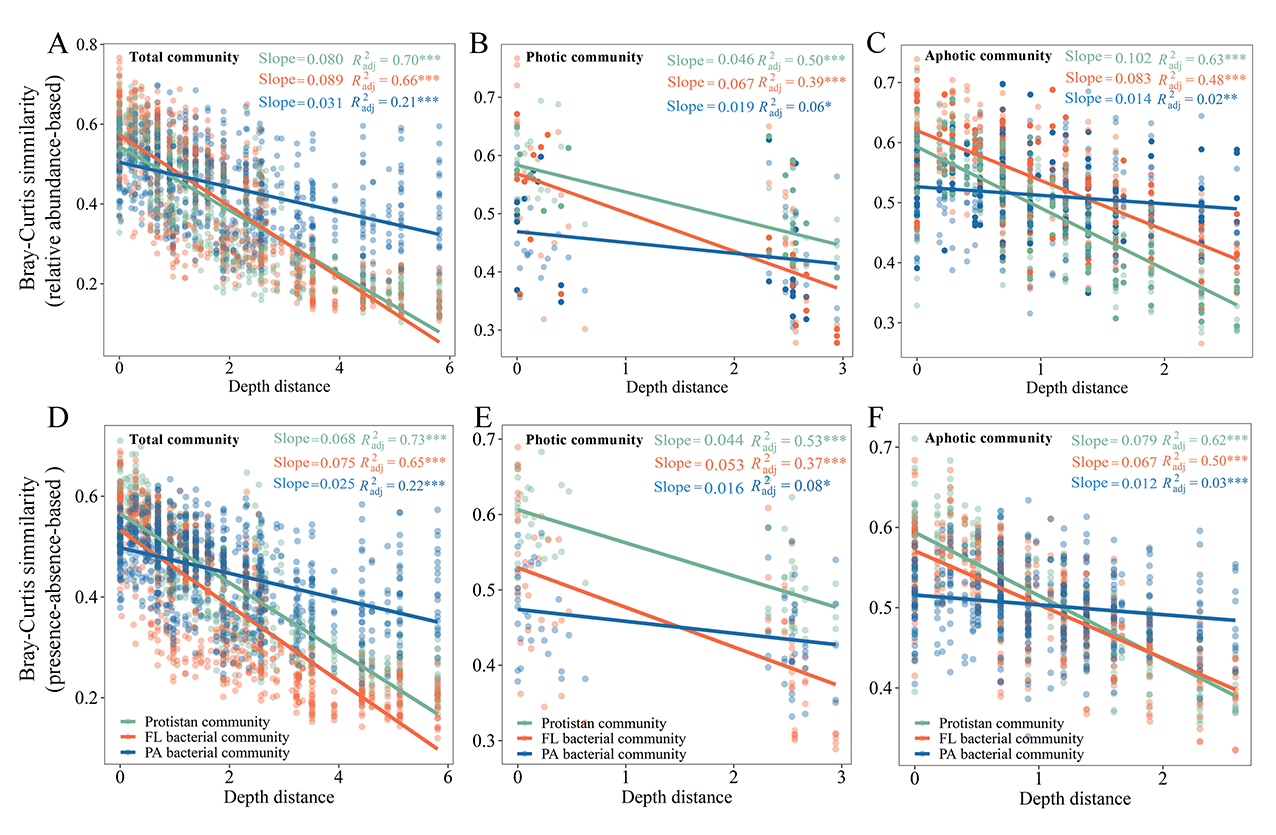

Supplement: FIG S2 [file msystems.00013-23-s0002.tif]

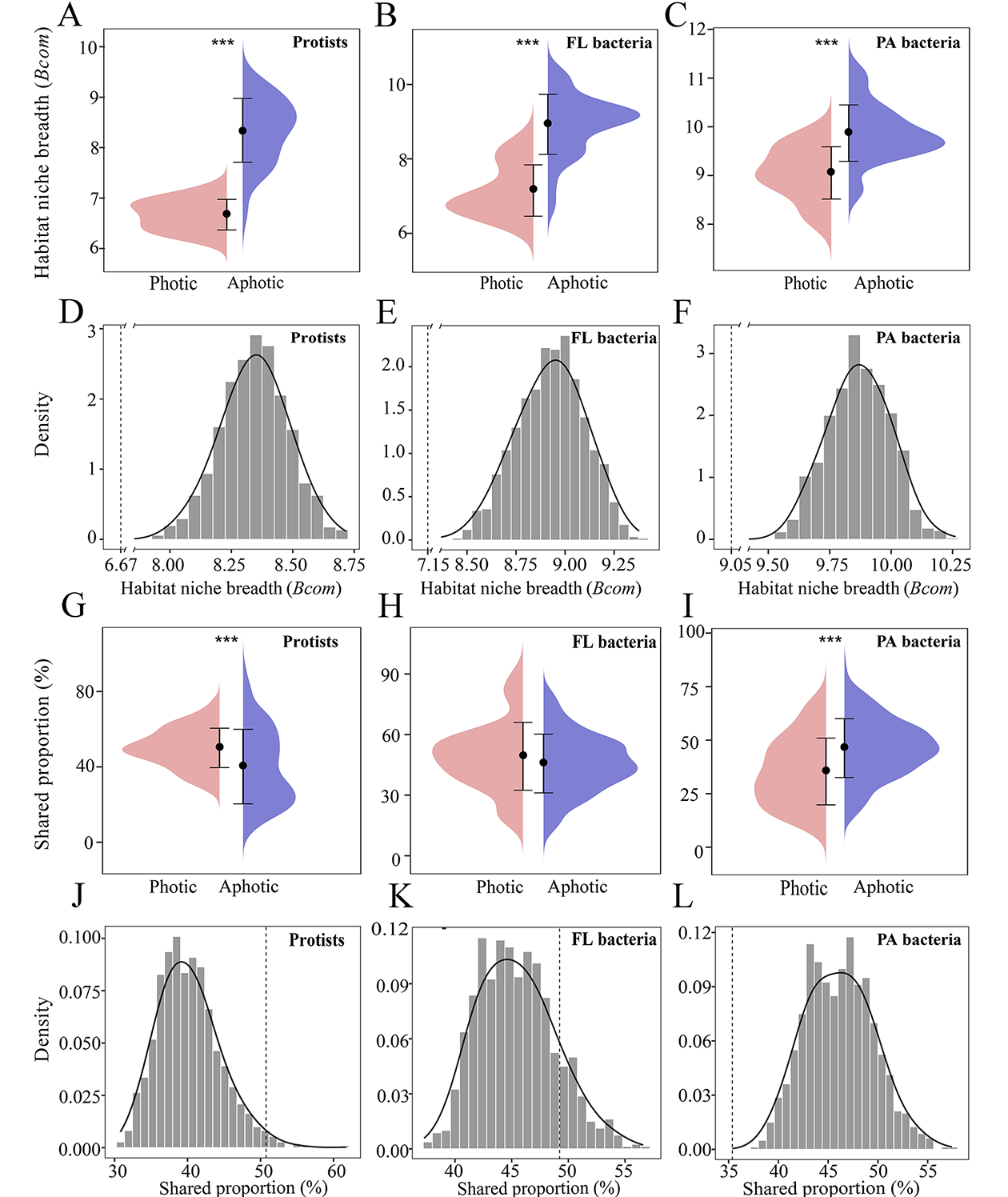

Supplement: FIG S3 [file msystems.00013-23-s0003.tif]

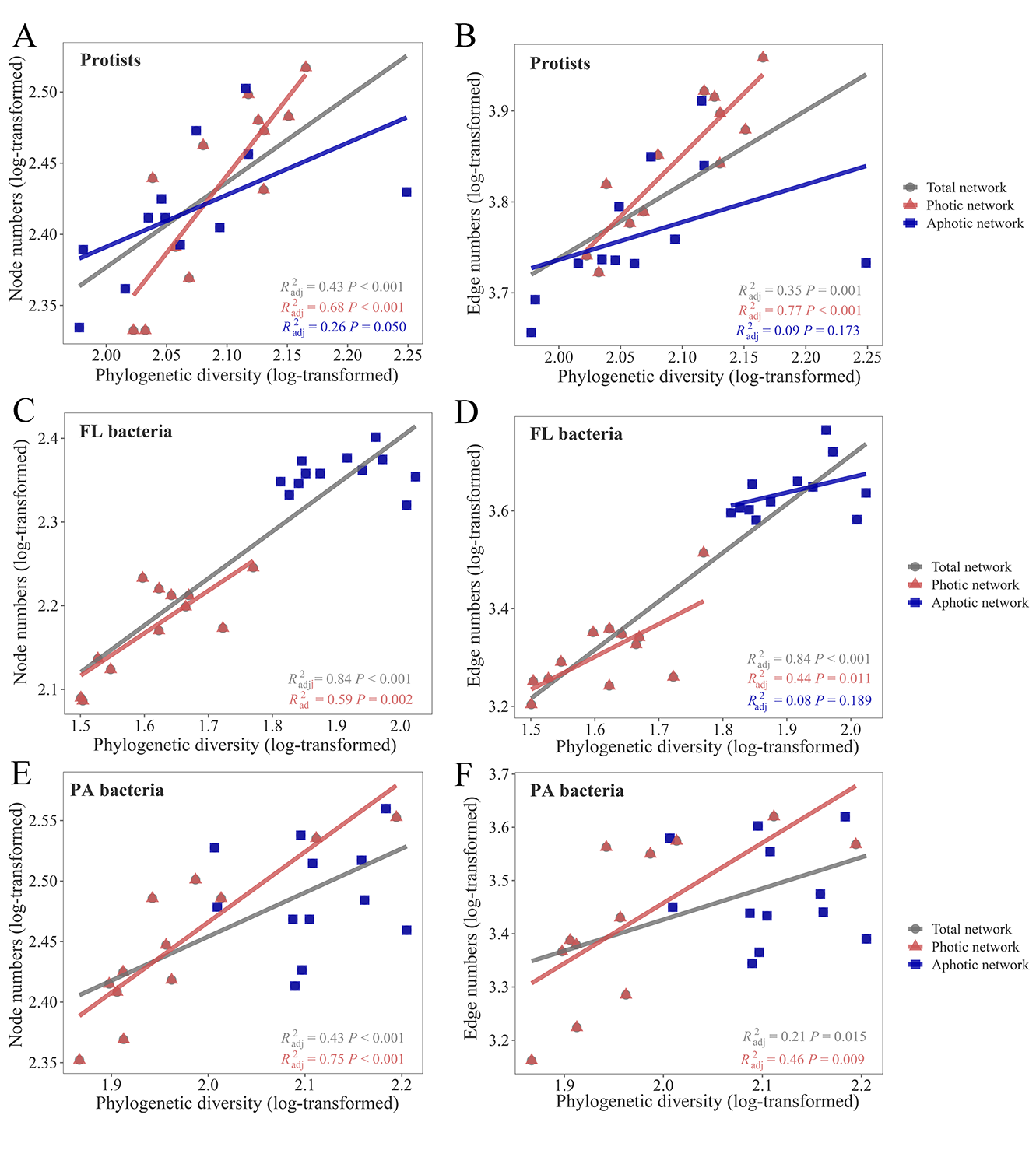

Supplement: FIG S4 [file msystems.00013-23-s0004.tif]

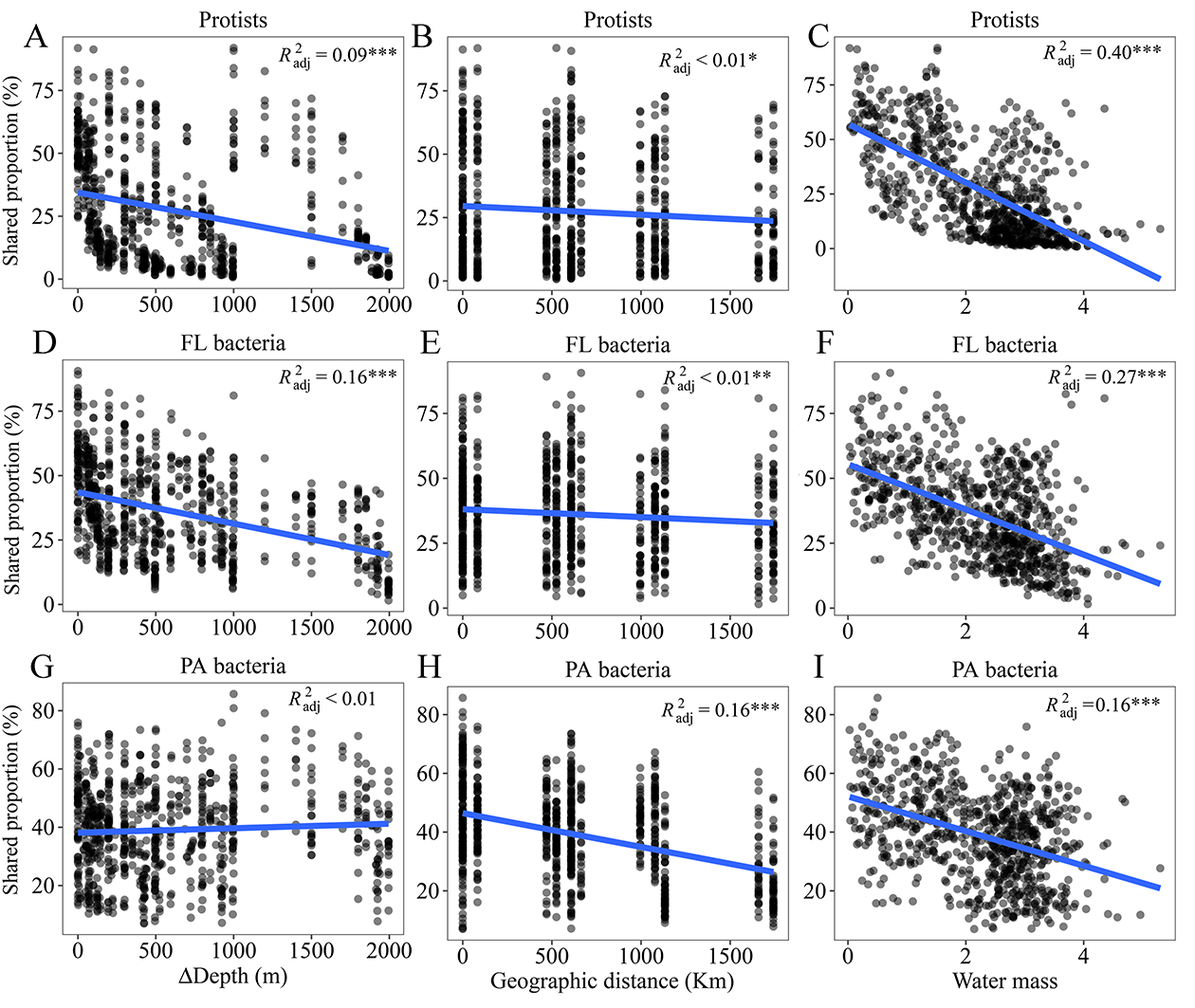

Supplement: FIG S5 [file msystems.00013-23-s0005.tif]
