# Supplementary material for: From the Sunlit to the Aphotic Zone: Assembly Mechanisms and Co-Occurrence Patterns of Protistan-Bacterial Microbiotas in the Western Pacific Ocean
Source: mSystems. 2023 Feb 27;8(2):e00013-23. doi: 10.1128/msystems.00013-23 (PMC10134807; doi:10.1128/msystems.00013-23)
Supplement: TABLE S1 [file msystems.00013-23-s0006.docx]

| Station | Longitude (°E) | Latitude(°N) | Sampling date | Depth (m) |
| --- | --- | --- | --- | --- |
| E130-7 | 130 | 18 | 2018-10-12 | 5 |
| E130-7 | 130 | 18 | 2018-10-12 | 77 |
| E130-7 | 130 | 18 | 2018-10-12 | 200 |
| E130-7 | 130 | 18 | 2018-10-12 | 500 |
| E130-7 | 130 | 18 | 2018-10-12 | 1000 |
| E130-7 | 130 | 18 | 2018-10-12 | 2000 |
| E130-13 | 130 | 12 | 2018-10-15 | 5 |
| E130-13 | 130 | 12 | 2018-10-15 | 113 |
| E130-13 | 130 | 12 | 2018-10-15 | 200 |
| E130-13 | 130 | 12 | 2018-10-15 | 500 |
| E130-13 | 130 | 12 | 2018-10-15 | 1000 |
| E130-13 | 130 | 12 | 2018-10-15 | 2000 |
| E130-28 | 130 | 3 | 2018-10-22 | 5 |
| E130-28 | 130 | 3 | 2018-10-22 | 70 |
| E130-28 | 130 | 3 | 2018-10-22 | 150 |
| E130-28 | 130 | 3 | 2018-10-22 | 200 |
| E130-28 | 130 | 3 | 2018-10-22 | 500 |
| E130-28 | 130 | 3 | 2018-10-22 | 1000 |
| E130-28 | 130 | 3 | 2018-10-22 | 2000 |
| ME-3 | 130 | 7.75 | 2018-10-17 | 5 |
| ME-3 | 130 | 7.75 | 2018-10-17 | 75 |
| ME-3 | 130 | 7.75 | 2018-10-17 | 85 |
| ME-3 | 130 | 7.75 | 2018-10-17 | 150 |
| ME-3 | 130 | 7.75 | 2018-10-17 | 200 |
| ME-3 | 130 | 7.75 | 2018-10-17 | 300 |
| ME-3 | 130 | 7.75 | 2018-10-17 | 500 |
| ME-3 | 130 | 7.75 | 2018-10-17 | 600 |
| ME-3 | 130 | 7.75 | 2018-10-17 | 800 |
| ME-3 | 130 | 7.75 | 2018-10-17 | 1000 |
| ME-3 | 130 | 7.75 | 2018-10-17 | 2000 |
| ME-14 | 130 | 2.25 | 2018-10-23 | 5 |
| ME-14 | 130 | 2.25 | 2018-10-23 | 75 |
| ME-14 | 130 | 2.25 | 2018-10-23 | 60 |
| ME-14 | 130 | 2.25 | 2018-10-23 | 150 |
| ME-14 | 130 | 2.25 | 2018-10-23 | 200 |
| ME-14 | 130 | 2.25 | 2018-10-23 | 300 |
| ME-14 | 130 | 2.25 | 2018-10-23 | 500 |
| ME-14 | 130 | 2.25 | 2018-10-23 | 600 |
| ME-14 | 130 | 2.25 | 2018-10-23 | 800 |
| ME-14 | 130 | 2.25 | 2018-10-23 | 1000 |
| ME-14 | 130 | 2.25 | 2018-10-23 | 2000 |

**Table S1**. Sampling information of waters collected from the western Pacific Oceans.
