# Supplementary material for: From the Sunlit to the Aphotic Zone: Assembly Mechanisms and Co-Occurrence Patterns of Protistan-Bacterial Microbiotas in the Western Pacific Ocean
Source: mSystems. 2023 Feb 27;8(2):e00013-23. doi: 10.1128/msystems.00013-23 (PMC10134807; doi:10.1128/msystems.00013-23)
Supplement: TABLE S2 [file msystems.00013-23-s0007.docx]

**Table S2.** Phylogenetic null model-based mapping of ecological processes in protistan, FL-, and PA-bacterial communities in photic and aphotic zones.

| Ecological  process | | Stochastic processes (%) | Deterministic processes (%) | Stochastic processes /Deterministic processes |
| --- | --- | --- | --- | --- |
| Protist | photic | 18.18 | 12.12 | 1.500 |
|  | aphotic | 42.33 | 22.22 | 1.905 |
| FL bacteria | photic | 9.09 | 45.45 | 0.200 |
|  | aphotic | 31.53 | 28.57 | 1.103 |
| PA bacteria | photic | 12.12 | 37.88 | 0.320 |
|  | aphotic | 19.21 | 24.63 | 0.780 |

Stochastic processes are dispersal limitation and homogenizing dispersal.

Deterministic processes are heterogeneous selection and homogeneous selection.
