# Supplementary material for: From the Sunlit to the Aphotic Zone: Assembly Mechanisms and Co-Occurrence Patterns of Protistan-Bacterial Microbiotas in the Western Pacific Ocean
Source: mSystems. 2023 Feb 27;8(2):e00013-23. doi: 10.1128/msystems.00013-23 (PMC10134807; doi:10.1128/msystems.00013-23)
Supplement: TABLE S3 [file msystems.00013-23-s0008.docx]

**Table S3**. Topological properties of the empirical co-occurrence networks and associated random networks in photic and aphotic zones.

| Network properties | | Photic network | Aphotic network |
| --- | --- | --- | --- |
| Empirical networks | Node | 986 | 1050 |
|  | Edge | 8172 | 5207 |
|  | Average degree | 16.576 | 9.918 |
|  | Network diameter | 14 | 13 |
|  | Network density | 0.017 | 0.009 |
|  | Modularity | 0.45 | 0.516 |
|  | Average clustering coefficient | 0.282 | 0.200 |
|  | Average path length | 4.083 | 4.376 |
|  | R^2^ of power-law | 0.811 | 0.862 |
| Random networks | Modularity | 0.172 (±0.004) | 0.232 (±0.006) |
|  | Average clustering coefficient | 0.017 (±0.001) | 0.009 (±0.001) |
|  | Average path length | 2.748 (±0.001) | 3.289 (±0.002) |
|  | Small-word coefficient (σ) | 11.285 (±0.416) | 15.992 (±1.2434 |
