# Supplementary material for: From the Sunlit to the Aphotic Zone: Assembly Mechanisms and Co-Occurrence Patterns of Protistan-Bacterial Microbiotas in the Western Pacific Ocean
Source: mSystems. 2023 Feb 27;8(2):e00013-23. doi: 10.1128/msystems.00013-23 (PMC10134807; doi:10.1128/msystems.00013-23)
Supplement: TABLE S4 [file msystems.00013-23-s0009.docx]

**Table S4**. The regression coefficient between environmental variables and network size (nodes) and network connectivity (edges).

|  | Node numbers | | | | | | Edge numbers | | | | | |
| --- | --- | --- | --- | --- | --- | --- | --- | --- | --- | --- | --- | --- |
|  | Total network | | Photic network | | Aphotic network | | Total network | | Photic network | | Aphotic network | |
| Protists | *R^2^* | *P* | *R^2^* | *P* | *R^2^* | *P* | *R^2^* | *P* | *R^2^* | *P* | *R^2^* | *P* |
| HNF biomass (2-5 μm) | <0.01 | 0.914 | <0.01 | 0.434 | **0.35** | **0.024** | 0.03 | 0.213 | <0.01 | 0.514 | **0.44** | **0.011** |
| HNF biomass (5-10 μm) | <0.01 | 0.849 | <0.01 | 0.646 | <0.01 | 0.438 | 0.05 | 0.152 | <0.01 | 0.959 | <0.01 | 0.505 |
| NF biomass (2-5 μm) | 0.08 | 0.099 | 0.07 | 0.201 | **0.53** | **0.004** | **0.32** | **0.002** | 0.08 | 0.189 | **0.62** | **0.001** |
| NF biomass (5-10 μm) | 0.02 | 0.221 | <0.01 | 0.580 | **0.38** | **0.019** | **0.26** | **0.006** | <0.01 | 0.429 | **0.40** | **0.016** |
| Dissolved oxygen | <0.01 | 0.774 | <0.01 | 0.999 | 0.03 | 0.278 | <0.01 | 0.852 | <0.01 | 0.899 | <0.01 | 0.386 |
| Salinity | **0.35** | **0.001** | **0.51** | **0.005** | 0.26 | 0.051 | 0.12 | 0.056 | **0.43** | **0.012** | 0.20 | 0.083 |
| Temperature | 0.02 | 0.222 | 0.09 | 0.180 | **0.59** | **0.002** | **0.31** | **0.003** | 0.02 | 0.286 | **0.63** | **0.001** |
| Viral abundance | 0.06 | 0.136 | <0.01 | 0.555 | **0.17** | **0.099** | **0.27** | **0.006** | <0.01 | 0.776 | 0.22 | 0.071 |
| FL bacteria |  |  |  |  |  |  |  |  |  |  |  |  |
| HNF biomass (2-5 μm) | **0.35** | **0.001** | 0.08 | 0.189 | <0.01 | 0.783 | **0.34** | **0.002** | 0.06 | 0.225 | <0.01 | 0.732 |
| HNF biomass (5-10 μm) | **0.32** | **0.002** | 0.14 | 0.126 | 0.01 | 0.312 | **0.21** | **0.013** | <0.01 | 0.583 | <0.01 | 0.352 |
| NF biomass (2-5 μm) | **0.51** | **0.001** | <0.01 | 0.333 | <0.01 | 0.969 | **0.51** | **0.001** | 0.10 | 0.163 | <0.01 | 0.366 |
| NF biomass (5-10 μm) | **0.54** | **0.010** | <0.01 | 0.674 | <0.01 | 0.432 | **0.49** | **0.001** | 0.13 | 0.139 | <0.01 | 0.958 |
| Dissolved oxygen | <0.01 | 0.383 | <0.01 | 0.803 | 0.03 | 0.277 | <0.01 | 0.686 | <0.01 | 0.645 | 0.13 | 0.136 |
| Salinity | 0.11 | 0.067 | 0.23 | 0.065 | <0.01 | 0.318 | 0.03 | 0.211 | 0.06 | 0.228 | **0.35** | **0.025** |
| Temperature | **0.73** | **0.001** | 0.09 | 0.178 | <0.01 | 0.863 | **0.78** | **0.001** | 0.04 | 0.256 | 0.07 | 0.204 |
| Viral abundance | **0.50** | **0.001** | <0.01 | 0.581 | <0.01 | 0.792 | **0.61** | **0.001** | <0.01 | 0.619 | 0.01 | 0.480 |
| PA bacteria |  |  |  |  |  |  |  |  |  |  |  |  |
| HNF biomass (2-5 μm) | <0.01 | 0.813 | 0.04 | 0.255 | <0.01 | 0.644 | <0.01 | 0.460 | 0.07 | 0.202 | <0.01 | 0.968 |
| HNF biomass (5-10 μm) | <0.01 | 0.285 | 0.26 | 0.051 | 0.01 | 0.312 | 0.10 | 0.075 | **0.38** | **0.019** | <0.01 | 0.497 |
| NF biomass (2-5 μm) | 0.02 | 0.229 | <0.01 | 0.781 | <0.01 | 0.780 | <0.01 | 0.576 | <0.01 | 0.711 | <0.01 | 0.945 |
| NF biomass (5-10 μm) | <0.01 | 0.302 | <0.01 | 0.630 | <0.01 | 0.560 | <0.01 | 0.770 | <0.01 | 0.467 | <0.01 | 0.775 |
| Dissolved oxygen | <0.01 | 0.710 | <0.01 | 0.833 | <0.01 | 0.506 | <0.01 | 0.872 | <0.01 | 0.848 | <0.01 | 0.622 |
| Salinity | <0.01 | 0.933 | <0.01 | 0.955 | <0.01 | 0.414 | <0.01 | 0.530 | <0.01 | 0.622 | <0.01 | 0.353 |
| Temperature | 0.02 | 0.223 | <0.01 | 0.354 | <0.01 | 0.939 | <0.01 | 0.565 | 0.09 | 0.174 | <0.01 | 0.780 |
| Viral abundance | 0.05 | 0.158 | <0.01 | 0.489 | <0.01 | 0.393 | 0.03 | 0.191 | 0.10 | 0.168 | <0.01 | 0.535 |

HNF, heterotrophic nanoflagellate; NF, nanoflagellate.
