# Supplementary material for: From the Sunlit to the Aphotic Zone: Assembly Mechanisms and Co-Occurrence Patterns of Protistan-Bacterial Microbiotas in the Western Pacific Ocean
Source: mSystems. 2023 Feb 27;8(2):e00013-23. doi: 10.1128/msystems.00013-23 (PMC10134807; doi:10.1128/msystems.00013-23)
Supplement: TABLE S5 [file msystems.00013-23-s0010.docx]

**Table S5**. List of the keystone OTUs identified in both photic and aphotic networks.

| OTU ID | Taxonomy |
| --- | --- |
| Protists |  |
| Otu174 | Eukaryota; Alveolata; Dinophyta; Syndiniales; Dino-Group-III; Dino-Group-III_X; Dino-Group-III_XX; Dino-Group-III_XX_sp. |
| Otu275 | Eukaryota; Hacrobia; Centroheliozoa; Centroheliozoa_X; Pterocystida; Pterocystida_X; Pterocystida_XX; Pterocystida_XX_sp. |
| FL bacteria |  |
| Otu115 | Bacteria; Proteobacteria; Alphaproteobacteria; Rhodospirillales; Thalassospiraceae; Thalassospira; alpha proteobacterium B-1121 |
| Otu2662 | Bacteria; Proteobacteria; Alphaproteobacteria; SAR11 clade; Clade I; uncultured; uncultured bacterium |
| Otu4726 | Bacteria; Proteobacteria; Alphaproteobacteria; SAR11 clade; Clade I; uncultured; uncultured bacterium |
| Otu64 | Bacteria; Actinobacteria; Acidimicrobiia; Microtrichales; Microtrichaceae; Sva0996 marine group; uncultured bacterium |
| PA bacteria |  |
| Otu218 | Bacteria; Actinobacteria; Acidimicrobiia; Microtrichales; Ilumatobacteraceae; Ilumatobacter; uncultured bacterium |
| Otu201 | Bacteria; Actinobacteria; Actinobacteria; Corynebacteriales; Mycobacteriaceae; Mycobacterium; Mycobacterium sp. JC422 |
| Otu353 | Bacteria; Bacteroidetes; Bacteroidia; Flavobacteriales; Flavobacteriaceae; Aureicoccus; uncultured bacterium |
| Otu30 | Bacteria; Cyanobacteria; Oxyphotobacteria; Nostocales; Phormidiaceae; Trichodesmium IMS101; Trichodesmium thiebautii SERB 30 |
| Otu1682 | Bacteria; Proteobacteria; Alphaproteobacteria; Caulobacterales; Caulobacteraceae; Brevundimonas; Brevundimonas sp. PP32 |
| Otu638 | Bacteria; Proteobacteria; Alphaproteobacteria; Caulobacterales; Parvularculaceae; Amphiplicatus; uncultured bacterium |
| Otu67 | Bacteria; Proteobacteria; Alphaproteobacteria; Rhodospirillales; Thalassospiraceae; Thalassospira; uncultured bacterium |
| Otu618 | Bacteria; Proteobacteria; Deltaproteobacteria; NB1-j; uncultured bacterium |
| Otu1089 | Bacteria; Proteobacteria; Gammaproteobacteria; Alteromonadales; Alteromonadaceae; Salinimonas; Salinimonas sp. TB125_2 |
| Otu122 | Bacteria; Verrucomicrobia; Verrucomicrobiae; Verrucomicrobiales; DEV007; uncultured bacterium |
